# Influenza A virus use of BinCARD1 to facilitate the binding of viral NP to importin α7 is counteracted by TBK1-p62 axis-mediated autophagy

**DOI:** 10.1038/s41423-022-00906-w

**Published:** 2022-09-02

**Authors:** Xuyuan Wang, Li Jiang, Guangwen Wang, Wenjun Shi, Yuzhen Hu, Bo Wang, Xianying Zeng, Guobin Tian, Guohua Deng, Jianzhong Shi, Liling Liu, Chengjun Li, Hualan Chen

**Affiliations:** 1 College of Veterinary Medicine, Gansu Agricultural University, Lanzhou, 730070 China; 2 Guangdong Laboratory for Lingnan Modern Agriculture, Guangzhou, 510642 China; 3 State Key Laboratory of Veterinary Biotechnology, Harbin Veterinary Research Institute, Chinese Academy of Agricultural Sciences, Harbin, 150069 China

**Keywords:** Influenza A virus, BinCARD1, TRAF3, TBK1, p62, Signal transduction, Infectious diseases

## Abstract

As a major component of the viral ribonucleoprotein (vRNP) complex in influenza A virus (IAV), nucleoprotein (NP) interacts with isoforms of importin α family members, leading to the import of itself  and vRNP complex into the nucleus, a process pivotal in the replication cycle of IAV. In this study, we found that BinCARD1, an isoform of Bcl10-interacting protein with CARD (BinCARD), was leveraged by IAV for efficient viral replication. BinCARD1 promoted the nuclear import of the vRNP complex and newly synthesized NP and thus enhanced vRNP complex activity. Moreover, we found that BinCARD1 interacted with NP to promote NP binding to importin α7, an adaptor in the host nuclear import pathway. However, we also found that BinCARD1 promoted RIG-I-mediated innate immune signaling by mediating Lys63-linked polyubiquitination of TRAF3, and that TBK1 appeared to degrade BinCARD1. We showed that BinCARD1 was polyubiquitinated at residue K103 through a Lys63 linkage, which was recognized by the TBK1-p62 axis for autophagic degradation. Overall, our data demonstrate that IAV leverages BinCARD1 as an important host factor that promotes viral replication, and two mechanisms in the host defense system are triggered—innate immune signaling and autophagic degradation—to mitigate the promoting effect of BinCARD1 on the life cycle of IAV.

## Introduction

Influenza A virus (IAV) belongs to the family Orthomyxoviridae. The genome of IAV consists of eight single-stranded negative-sense RNA segments. Each viral RNA (vRNA) segment terminus associates with an RNA-dependent RNA polymerase composed of three polymerase proteins [polymerase basic protein 1 (PB1), polymerase basic protein 2 (PB2), and polymerase acidic protein (PA)], with the remainder of the segment wrapping around the nucleoprotein (NP) strands [1–3], forming the vRNP complex.

The vRNP complex plays a central role in the life cycle of IAV by catalyzing the transcription and replication of the viral genome [4, 5] in the nucleus of infected cells [6]. Because it is large, the vRNP complex is unable to enter the nucleus through passive diffusion. Upon release from uncoated invading virions, the vRNP complex is imported into the nucleus via the classical nuclear import pathway, a translocation process similar to that of many nuclear proteins. In this pathway, the vRNP complex relies on NP for nuclear import, which is largely dependent on the binding between the nuclear localization signals (NLSs) in NP and isoforms of importin α (IMP α) and the binding between IMP α and importin β1 (IMP β1) [6–11]. Two NLSs have been identified in NP: an unconventional NLS in the N-terminus (NLS1; residues 3 to 13) [12, 13] and a bipartite NLS (NLS2; residues 198 to 216) [14]. NLS1 plays a major role in the nuclear import of vRNPs, although it binds weakly to the NLS-binding domain of IMP α [15]. NP interacts with various isoforms of IMP α, including IMP α-1, -3, -5, and -7 [16, 17]. After the recognition of the NLSs in NP by IMP α family members, IMP β1 is recruited to form a trimer that enters the nucleus mediated by nucleoporins [18, 19]. In addition to the central role played by the IMP α/β complex in the nuclear transport of the vRNP complex, several host factors have been shown to participate in the regulation of the nuclear import of the vRNP complex and/or newly synthesized NP, such as PLSCR1, MOV10, HAX1, eEF1D, and Hsp40/DnaJB1 [17, 20–23]. However, given the importance of the vRNP complex in the IAV life cycle, additional, as-yet undiscovered novel host factors must be involved in vRNP nuclear import.

The host factors that facilitate IAV replication can encounter resistance from the host defense system. The innate immune system is the first line of host defense against viral infection. The retinoic acid-inducible gene I (RIG-I) innate immune signaling pathway plays an important antiviral role to hinder IAV infection. In the cytoplasm, RIG-I senses the 5’ppp-dsRNA panhandle structure that is formed at the ends of viral RNA. Upon binding of the CTD domain to viral RNA [24], RIG-I undergoes conformational changes and oligomerization, exposing the CARD domains to recruit a signaling adaptor called mitochondrial antiviral-signaling protein (MAVS). Acting as the docking site, MAVS recruits the downstream TNF receptor associated factor 3 (TRAF3), tank binding kinase 1 (TBK1), and inhibitor of kappa B kinase ε (IKKε), resulting in the activation of interferon regulatory factor 3 (IRF3) [25–27]. Activated IRF3 is then translocated from the cytoplasm to the nucleus and associates with interferon-stimulated response elements (ISREs) in the promoter of interferon-beta (IFN-β) and interferon-alpha (IFN-α), leading to the production of type I IFNs [24, 28–31]. TBK1 not only plays an important role in the type I IFN production pathway but also phosphorylates and activates autophagy receptors such as p62/SQSTM1 and OPTN, thereby promoting autophagy [31–36].

As a member of the caspase recruitment domain family, Bcl10-interacting protein with CARD (BinCARD) is mainly expressed as two isoforms, BinCARD1 and BinCARD2 [37]. The N-terminal structures of both isoforms are essentially identical, with both including a CARD domain; the C-terminus of BinCARD2 also includes a transmembrane domain, which is required for its localization to the endoplasmic reticulum (ER) and mitochondria [37]. Functionally, BinCARD2 positively regulates the RIG-I innate immune response by interacting with and promoting the oligomerization of MAVS, and BinCARD1 inhibits the NFκB pathway by inhibiting the phosphorylation of Bcl10 [38, 39]. However, the role played by BinCARD in the replication cycle of IAV is unknown, and the regulation of its function has not been reported, which prompted us to undertake this study.

## Materials and methods

### Cells and viruses

Human lung carcinoma cells (A549), human embryonic kidney cells (HEK293T), Madin-Darby canine kidney cells (MDCK), and murine macrophage cells (RAW264.7) were cultured at 37 °C in a humidified 5% CO_2_ incubator in F-12K medium (Life Technologies) supplemented with 10% fetal bovine serum (FBS; Sigma‒Aldrich), Dulbecco’s modified Eagle’s medium (DMEM; Life Technologies) supplemented with 10% FBS, DMEM supplemented with 6% newborn calf serum (NCS; Sigma‒Aldrich), and RPMI 1640 medium (Life Technologies) supplemented with 10% FBS, respectively. All media contained 100 units/ml penicillin and 100 μg/ml streptomycin (Life Technologies).

A/WSN/33 (WSN, H1N1) virus was propagated in MDCK cells cultured in MEM containing 0.3% bovine serum albumin (BSA, Sigma‒Aldrich) and 0.5 μg/ml L-1-tosylamide-2-phenylmethyl chloromethyl ketone (TPCK)-treated trypsin (Sigma‒Aldrich). A/Anhui/2/2005 (AH05, H5N1) and A/Anhui/1/2013 (AH13, H7N9) were cultured in 10-day-old embryonated chicken eggs. Sendai virus (SeV) was provided by Dr. Jin Tian [Harbin Veterinary Research Institute (HVRI), Chinese Academy of Agricultural Sciences (CAAS), China].

All experiments with live H5N1 and H7N9 viruses were conducted in the enhanced animal biosafety level 3 (ABSL3+) facility at HVRI, CAAS, which was approved for use by the Ministry of Agriculture and Rural Affairs of the People’s Republic of China and the China National Accreditation Service for Conformity Assessment.

### Antibodies

The following primary antibodies were obtained from commercial sources: mouse anti-BinCARD1 (102-228 aa) mAb (Beijing Protein Innovation), rabbit anti-BinCARD pAb (PA5-89068, Thermo Fisher Scientific), mouse anti-Flag mAb (F3165, Sigma‒Aldrich), rabbit anti-Flag pAb (F7425, Sigma‒Aldrich), rabbit anti-HA mAb (3724, Cell Signaling Technology), rabbit anti-GAPDH pAb (10494-1-AP, Proteintech), mouse anti-GST mAb (A00865, GenScript), rabbit anti-GST pAb (A00097, GenScript), rabbit anti-LaminB1 pAb (12987-1-AP, Proteintech), mouse anti-Myc mAb (M4439, Sigma‒Aldrich), rabbit anti-Myc pAb (C3956, Sigma‒Aldrich), mouse anti-TRAF3 mAb (sc-6933, Santa Cruz), rabbit anti-MAVS mAb (83000, Cell Signaling Technology), mouse anti-p62 mAb (88588, Cell Signaling Technology), rabbit anti-phospho-SQSTM1/p62 (Ser403) mAb (39786, Cell Signaling Technology), rabbit anti-IRF3-p mAb (29047, Cell Signaling Technology), rabbit anti-TBK1 mAb (3504, Cell Signaling Technology), rabbit anti-LC3B pAb (2775, Cell Signaling Technology), rabbit anti-M1 pAb (GTX125928, GeneTex), mouse anti-ubiquitin mAb (sc-8017, Santa Cruz), and rabbit anti-ubiquitin (linkage-specific K63) mAb (ab179434, Abcam). The mouse anti-PB2, PB1, PA and NP mAbs were produced as previously described [40]. The rabbit anti-NP pAb was produced and stored in our laboratory. The secondary antibodies used for western blotting were DyLight 800 goat anti-mouse IgG (H + L) (RS23910) and DyLight 800 goat anti-rabbit IgG (H + L) (RS23920), purchased from ImmunoWay; the secondary antibodies used for confocal microscopy were Alexa Fluor 488 goat anti-mouse IgG (H + L) (A11029), Alexa Fluor 488 goat anti-rabbit IgG (H + L) (A11034), Alexa Fluor 633 goat anti-mouse IgG (H + L) (A21052) and Alexa Fluor 633 goat anti-rabbit IgG (H + L) (A21071), which were obtained from Life Technologies.

### Construction of plasmids

The open reading frames (ORFs) of the human BinCARD1 and BinCARD2 genes obtained from total cellular mRNAs of A549 cells were amplified by RT‒PCR and cloned into the pCAGGS vector with a Flag, GST or Myc tag at the N-terminus or C-terminus. The pCAGGS plasmids bearing the ORFs of the PB2, PB1, PA, and NP genes of WSN (H1N1) virus as well as truncation mutants of GST-tagged WSN NP were constructed as described previously [20]. The construction of pHH21-SC09NS F-Luc, which was used to produce an influenza vRNA-like luciferase reporter, and an ISRE-Luc reporter has been previously described [20, 41]. The ORFs of human IMP α1, IMP α3, IMP α5, IMP α7, and IMP β1 with a Flag tag at the N-terminus were cloned into a pCAGGS vector. The pCAGGS constructs bearing a Flag tag in the C-terminus of the ORFs of human RIG-I, MAVS, TRAF3, TBK1, IKKε and IRF3 were generated as described previously [41]. The ORFs of human OPTN and p62/SQSTM1 with a Myc tag at the C-terminus were cloned into pCAGGS, and the ORF of human ubiquitin was cloned into pCAGGS with an HA tag in the N-terminus. Truncation mutants of GST-BinCARD1 and TBK1-Flag were generated using a PCR approach and cloned into pCAGGS. Point mutants of GST-BinCARD1, HA-tagged ubiquitin, and TRAF3-Flag were generated using the Fast Mutagenesis System (Transgen). All plasmid constructs were confirmed by sequencing.

### siRNA treatment and virus infection

siRNA targeting BinCARD, BinCARD2, or scrambled siRNA was transfected at a concentration of 30 nM with the Lipofectamine RNAiMAX transfection reagent (Invitrogen) into A549 cells cultured in 12-well plates. At 36 h post-transfection (p.t.), the knockdown efficiency was determined by qRT‒PCR, confocal microscopy or western blotting. The siRNA-treated A549 cells were infected with WSN (H1N1) (MOI = 0.01), AH05 (H5N1) (MOI = 0.1), or AH13 (H7N9) (MOI = 0.1) viruses. Supernatants were collected at the indicated timepoints, and virus titers were determined by plaque assays in MDCK cells.

### Generation of BinCARD_KO A549 cells and virus infection

BinCARD_KO A549 cells were established with the CRISPR/Cas9 system. The BinCARD gene target sequences, 5’-CACCGGTGGGTAGTAACGGTTCAGC-3’ and 5’-CACCGCTGCACCAGGCGGTCACAAT-3’, were inserted into a pSpCas9(BB)-2A-GFP (pX458) vector, which harbored a cassette for expressing Cas9 and EGFP. Six micrograms of each pX458 construct carrying one of the BinCARD targeting sequences was coelectrotransfected into A549 cells. Electrotransfected cells were trypsinized 48 h later, and single cells were then seeded into each well of a 96-well plate and subjected to fluorescence-activated cell sorting (FACS) with a SH800S Cell Sorter (Sony Biotechnology). Knockout of BinCARD expression was confirmed by sequencing, confocal microscopy, and western blotting. BinCARD_KO A549 cells or control cells were infected with WSN (H1N1) (MOI = 0.01), AH05 (H5N1) (MOI = 0.1), or AH13 (H7N9) (MOI = 0.1) virus. Supernatants were collected at 24 and 48 h p.i., and virus titers were determined by plaque assays in MDCK cells.

### Generation of BinCARD1- and BinCARD2-overexpressing A549 cell lines and virus infection

The human BinCARD1 and BinCARD2 genes were cloned into a pLVX-ISRE-ZsGreen1 vector (Clontech). HEK293T cells cultured in 10-cm dishes were transfected with pLVX-BinCARD1, pLVX-BinCARD2 or an empty pLVX vector with Lipofectamine LTX and Plus reagents (Invitrogen). At 48 h p.t., the lentiviruses produced were collected and used to infect A549 cells in the presence of 6 mg/mL polybrene. Three days later, the surviving cells were sorted on a SH800S Cell Sorter with ZsGreen used as a marker. The sorted cells were individually cloned into 12-well plates, propagated, and examined for BinCARD1 and BinCARD2 overexpression via western blotting analysis. The two overexpressing cell lines or control cells were infected with WSN (H1N1) virus (MOI = 0.01). Supernatants were collected at 24 and 48 h p.i., and virus titers were determined by plaque assays in MDCK cells.

### Plaque assay

Plaque assays were performed with MDCK cells in 12-well plates as described previously [42]. Briefly, MDCK cells infected with 10-fold serial dilutions of virus samples in 1 × MEM (0.3% BSA) were incubated at 37 °C for 1 h. The cells were then washed with PBS and covered with 1% SeaPlaque agarose (Lonza) in 1 × MEM containing 0.3% BSA and 0.5 μg/mL TPCK-treated trypsin. After a 48–72 h incubation, the cells were fixed with formalin, and the plaques were stained with 0.1% crystal violet and counted.

### Confocal microscopy

A549 cells subjected to the indicated treatments were fixed with 4% paraformaldehyde (PFA) for 30 min and permeabilized with 0.5% Triton X-100 in PBS for 15 min. The permeabilized cells were blocked with 5% BSA in PBS for 1 h and then incubated with the indicated primary antibodies at 4 °C overnight. The cells were washed three times with PBS and incubated with Alexa Fluor 488 goat anti-rabbit or mouse IgG (H + L) and/or Alexa Fluor 633 goat anti-mouse or rabbit IgG (H + L) for 1 h. After three washes, the cells were incubated with DAPI (4,6-diamidino-2-phenylindole; Thermo Fisher Scientific) for 15 min to stain the nuclei. Images were acquired with an LSM 800 confocal microscope with Airyscan (Zeiss).

### RNA quantification

Total RNA was extracted from siRNA-treated A549 cells or HEK293T cells at 36 h p.t. with an RNeasy kit (QIAGEN). The first-strand cDNAs were synthesized with an oligo(dT) primer and random 6-mers with a PrimeScript RT reagent kit with gDNA Eraser (TaKaRa). Real-time PCR was performed using SYBR premix Ex Taq II (TaKaRa). Relative BinCARD mRNA quantities were determined by using the comparative cycle-threshold method, with cellular GAPDH serving as the endogenous reference and scrambled siRNA-treated cells serving as the controls.

HEK293T cells were transfected with a plasmid expressing BinCARD1 or empty vector, and at 36 h p.t., the cells were stimulated with SeV (50 HAU/mL) or poly (I:C) (10 μg/mL) for 6 h. Total RNA was extracted and reverse transcribed as described above. Relative RNA quantities of ISG15 and OAS1 were determined by the comparative cycle-threshold method, with cellular GAPDH serving as the endogenous reference and empty vector transfected cells serving as the controls.

A549 cells were transfected with BinCARD siRNA1 or scrambled siRNA, and at 36 h p.t., the cells were infected with WSN (H1N1) virus (MOI = 5). At 3 and 6 h p.i., the levels of vRNA, mRNA, and cRNA derived from the NP gene were measured by RT‒qPCR as described previously [20].

### Cell viability assay

A CellTiter-Glo kit (Promega) was used to determine the cell viability as described previously [42]. Briefly, A549 cells seeded in opaque-walled 96-well plates were transfected with siRNA targeting BinCARD or BinCARD2 or with scrambled siRNA at a concentration of 30 nM. At 36 h p.t., 100 μl of CellTiter-Glo reagent was added directly to each well for 10 min to lyse the cells. Alternatively, BinCARD_KO A549 cells and A549 control cells were grown in opaque-walled 96-well plates until reaching confluency, and then cell lysates were prepared with CellTiter-Glo reagent. The luminescence of the cell lysates was measured with a GloMax 96 microplate luminometer.

### Dual-luciferase reporter assay

HEK293T cells treated for 36 h with siRNA targeting BinCARD or scrambled siRNA were transfected with pCAGGS constructs expressing the PB2, PB1, PA, and NP proteins (0.5 μg each) of WSN (H1N1) virus, the construct pHH21-SC09NS F-Luc (0.1 μg), and an internal control, pRL-TK (50 ng, Promega) using Lipofectamine LTX and Plus reagents (Invitrogen). In a separate experiment, HEK293T cells were transfected with pCAGGS constructs expressing the PB2, PB1, PA, and NP proteins of WSN (H1N1) virus and different amounts of BinCARD1-expressing plasmids, pHH21-SC09NS F-Luc, and pRL-TK. At 36 h p.t., cell lysates were prepared with a dual-luciferase reporter assay system (Promega), and the luciferase activities were measured on a GloMax 96 microplate luminometer (Promega). The cell lysates were subjected to western blotting to measure the expression levels of PB2, PB1, PA, and NP in the vRNP complex.

HEK293T cells were first transfected with an empty pCAGGS vector or pCAGGS-Flag-BinCARD1, the ISRE-Luc reporter plasmid (0.2 μg) and the pRL-TK control plasmid (0.01 μg). At 36 h p.t., the cells were either left untreated or stimulated with SeV (50 HAU/mL) or poly (I:C) (10 μg/mL) for 12 h. The overexpression of BinCARD1 was confirmed by western blotting with a rabbit anti-Flag pAb. The luciferase activity of the transfected cells was determined by performing a dual-luciferase reporter assay.

### Western blotting

Protein samples separated by SDS‒PAGE were transferred onto nitrocellulose membranes (GE Healthcare). The membranes were blocked with 5% skim milk in PBS at room temperature for 1 h, followed by incubation overnight at 4 °C with the appropriately diluted primary antibody in PBS containing 0.5% BSA. After further incubation at 4 °C for 1 h with DyLight 800 goat anti-rabbit IgG (H + L) and DyLight 800 goat anti-mouse IgG (H + L), the blots were visualized with an Odyssey CLX infrared imaging system (Li-Cor BioSciences).

### Co-IP assay

To examine interactions between proteins, HEK293T cells were transfected with the indicated plasmids with the Lipofectamine LTX and Plus Reagents (Invitrogen). Thirty-six hours later, the cells were washed with ice-cold PBS and lysed with IP lysis buffer (Pierce) that included a complete protease inhibitor cocktail (Roche Diagnostics GmbH) and PMSF for 30 min on ice. Cell lysates were then centrifuged at 12,000 rpm at 4 °C for 10 min. The supernatants were incubated with the indicated primary antibodies at 4 °C for 6–8 h, mixed with Protein G-Agarose beads (Roche) and rocked at 4 °C for 6–8 h. The beads were then washed three times with PBS containing 1% PMSF, and the bound proteins were separated by SDS‒PAGE and detected by western blotting.

### GST pull-down assay

HEK293T cells grown in 6-well plates were transfected with the indicated plasmids. At 36 h p.t., the cells were lysed with 250 μL of IP buffer. The lysates were pulled down with 8 μL of glutathione magnetic agarose (Invitrogen) for 8 h and then analyzed by western blotting.

### Cell fractionation

BinCARD_KO A549 cells and A549 control cells grown in 6-well plates were infected with WSN (H1N1) virus (MOI = 5). At 3 and 4 h p.i., the cells were separated into nuclear and cytoplasmic fractions with NE-PER Nuclear and Cytoplasmic Extraction Reagents (Pierce) according to the manufacturer’s instructions. The amount of NP in each fraction was determined by western blotting with a mouse anti-NP mAb. Lamin B1 and GAPDH, which are nuclear and cytoplasmic fraction markers, respectively, were detected by western blotting with a rabbit anti-GAPDH pAb and a rabbit anti-Lamin B1 pAb, respectively.

### Ubiquitination assay

HEK293T cells were transfected with the indicated plasmids, including constructs expressing HA-tagged ubiquitin or its mutants. At 36 h p.t., whole-cell lysate proteins were immunoprecipitated with the appropriate primary antibodies or incubated with glutathione magnetic agarose and analyzed by western blotting.

To determine whether BinCARD1 can induce K63-linked polyubiquitination of TRAF3 in infected cells, RAW264.7 cells were transfected with an empty vector or BinCARD1-expressing constructs, and at 48 h p.t., the cells were infected with SeV (50 HAU/mL). At 0 and 6 h p.i., whole-cell lysate proteins were immunoprecipitated with a mouse anti-TRAF3 mAb and analyzed by western blotting.

### Autophagy inhibitor and inducer treatments

To determine the pathway through which TBK1 degrades BinCARD1, HEK293T cells were first transfected to express BinCARD1, and 12 h later, the cells were transfected with an empty pCAGGS vector or pCAGGS-TBK1. Twenty-four hours later, the cells were treated with the proteasome inhibitor MG132 (8 mM) or the autophagy inhibitor 3-methyladenine (3-MA) (5 mg/mL) or bafilomycin A1 (Baf A1) (1 μM) for 8 h. Cell lysates were analyzed by western blotting with a mouse anti-BinCARD1 mAb and a rabbit anti-TBK1 mAb.

HEK293T cells were transfected with pCAGGS-BinCARD1, and at 24 h p.t., the autophagy inducers rapamycin (5 μM) and EBSS were added to stimulate the cells for 24 h and 2 h, respectively. Cell lysates were analyzed by western blotting with a mouse anti-BinCARD1 mAb.

### Statistical analysis

Statistical significance was determined by two-tailed unpaired Student’s *t* test or analysis of variance (ANOVA) test with GraphPad Prism 7.0 software. *P* values < 0.05 were considered to be statistically significant.

## Results

### IAV employs BinCARD1 for efficient replication

**Fig. 1:**
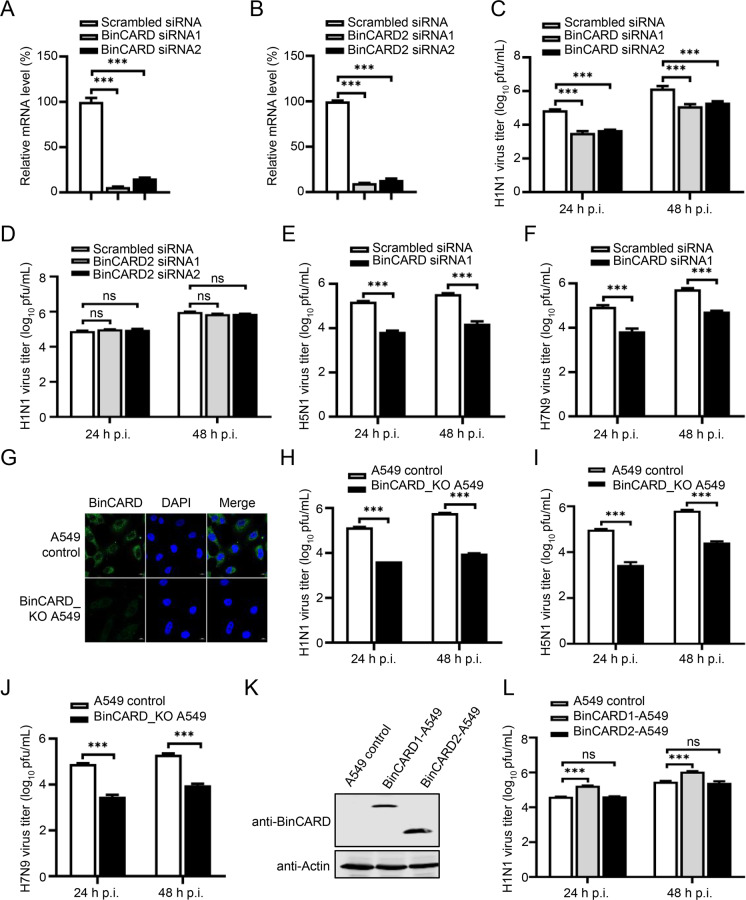
BinCARD1 promotes IAV replication. SiRNA knockdown of BinCARD and BinCARD2 in A549 cells. A549 cells were transfected with siRNA targeting BinCARD (**A**) or BinCARD2 (**B**) or with scrambled siRNA. At 36 h p.t., the knockdown efficiency was determined by RT‒qPCR. ***P < 0.001. Viral replication in siRNA-treated A549 cells. SiRNA-treated A549 cells are the same as those shown in **A**, **B** and were infected with WSN (H1N1) (MOI = 0.01) (**C**, **D**), AH05 (H5N1) (MOI = 0.1) (**E**), or AH13 (H7N9) (MOI = 0.1) (**F**) virus. Supernatants were collected at the indicated timepoints, and virus titers were determined by plaque assays in MDCK cells. ***P < 0.001; ns, not significant. **G** Knockout of BinCARD in BinCARD_KO A549 cells was confirmed by confocal microscopy with a rabbit anti-BinCARD pAb. Viral replication in BinCARD_KO A549 cells. BinCARD_KO A549 cells or A549 control cells were infected with WSN (H1N1) (MOI = 0.01) (**H**), AH05 (H5N1) (MOI = 0.1) (**I**), or AH13 (H7N9) (MOI = 0.1) (**J**) virus. Supernatants were collected at the indicated timepoints, and virus titers were determined by plaque assays in MDCK cells. ***P < 0.001. **K** Establishment of A549 cell lines stably overexpressing two isoforms of BinCARD. The stable lentivirus-mediated expression of BinCARD1 and BinCARD2 was confirmed by western blotting with a rabbit anti-BinCARD pAb. **L** Viral replication in BinCARD1- and BinCARD2-overexpressing A549 cells. BinCARD1- and BinCARD2-overexpressing A549 cells and A549 control cells are the same as those presented in **K** and were infected with WSN (H1N1) (MOI = 0.01) virus. Supernatants were collected at the indicated timepoints, and virus titers were determined by plaque assays in MDCK cells. ***P < 0.001. The data represent the mean ± SD of three independent experiments (**A**–**F**, **H**–**J**, **L**)

BinCARD was identified to be a potential proviral host factor for the replication of IAV in a previous whole-genome siRNA library screen performed with a replication-competent Venus-expressing H5N1 virus [43]. To determine whether BinCARD affects the replication of IAV, A549 cells were transfected with siRNA. For technical reasons, we designed only two siRNAs targeting the CCD region of the two BinCARD isoforms, as well as two siRNAs targeting BinCARD2. At 36 h p.t., the siRNA knockdown efficiency was verified by RT‒qPCR (Fig. 1A, B). Knocking down both BinCARD isoforms or only BinCARD2 with different siRNAs exerted no major effect on cell viability, as measured by a luminescent cell viability assay (Supplementary Fig. 1). The effect of siRNA knockdown of BinCARD on IAV replication was then evaluated after WSN (H1N1) virus infection. We found that when both BinCARD isoforms were simultaneously knocked down in A549 cells, the growth titers of WSN (H1N1) virus were reduced by 15- and 12-fold at 24 h post-infection (p.i.) and 8- and 4-fold at 48 h p.i. However, no significant changes in virus titers were observed between the A549 cells treated with siRNAs targeting BinCARD2 and those treated with scrambled siRNA (Fig. 1C, D). These results indicate that BinCARD1, but not BinCARD2, was required for the efficient replication of IAV. We also selected BinCARD siRNA1, whose downregulating effect on the expression of BinCARD/BinCARD1 at the protein level was confirmed by both confocal microscopy and western blotting (Supplementary Fig. 2A, B). We then examined the effect of reduced BinCARD/BinCARD1 protein levels on the levels of vRNA, mRNA, and cRNA of the NP gene in A549 cells infected with WSN (H1N1) virus. We found that the levels of all three species of viral RNA in the BinCARD siRNA1-treated cells were significantly lower than those in the scrambled siRNA-treated cells at both 3 h p.i. and 6 h p.i. (Supplementary Fig. 3A, B), thereby confirming that BinCARD1 was important for the efficient replication of WSN (H1N1) virus. In addition, the role played by BinCARD1 in the replication of IAV was validated by analyses performed with AH05 (H5N1) and AH13 (H7N9) viruses. We found that treatment with BinCARD siRNA1 in A549 cells led to AH05 (H5N1) and AH13 (H7N9) virus titer reductions of 23- and 13-fold at 24 h p.i. and 21- and 10-fold at 48 h p.i., respectively (Fig. 1E, F). These data demonstrate that BinCARD1 is required by a wide range of IAVs for efficient replication.

We next generated the BinCARD-knockout (BinCARD_KO) A549 cell line with the CRISPR/Cas9 system to confirm the positive regulatory effect of BinCARD1 on the replication of IAV. BinCARD/BinCARD1 knockout in BinCARD_KO A549 cells compared with that in control cells was confirmed by confocal microscopy and western blot analysis (Fig. 1G and Supplementary Fig. 4A). BinCARD knockout led to no major effect on cell viability (Supplementary Fig. 4B). The titers of WSN (H1N1) virus produced by BinCARD_KO A549 cells were 26- and 20-fold lower than those of control cells at 24 and 48 h p.i., respectively (Fig. 1H). Consistent with these data, 34-/24-fold and 27-/21-fold reductions in AH05 (H5N1) and AH13 (H7N9) virus titers were observed at 24/48 h p.i., respectively, in BinCARD_KO A549 cells compared with control cells (Fig. 1I, J).

To verify that BinCARD1 was solely responsible for the observed effect of BinCARD on IAV replication, we established BinCARD1-A549 and BinCARD2-A549 cell lines stably overexpressing BinCARD1 and BinCARD2, respectively, using a lentiviral system (Fig. 1K). We found that the titers of WSN (H1N1) virus in the BinCARD1-A549 cells were 5- and 4-fold higher at 24 and 48 h p.i. than those of the control cells. In contrast, overexpression of BinCARD2 in the BinCARD2-A549 cells did not affect the replication of WSN (H1N1) virus (Fig. 1L). Taken together, these data indicate that BinCARD1 positively regulated the replication of IAV.

To evaluate the biological significance of BinCARD1 in the replication cycle of IAV, we determined the expression profile of BinCARD1 in the course of IAV infection. A549 cells were infected with WSN (H1N1) virus (MOI = 0.01), and the level of BinCARD1 was measured by western blotting with a mouse anti-BinCARD1 mAb. We found that the level of BinCARD1 was gradually increased by infection with WSN (H1N1) virus. The level of BinCARD1 peaked at 24–36 h p.i. and then clearly decreased at 48 h p.i., suggesting that BinCARD1 function was important in the replication cycle of IAV (Supplementary Fig. 5).

### BinCARD1 positively regulates the nuclear import of the vRNP complex and newly synthesized NP

**Fig. 2:**
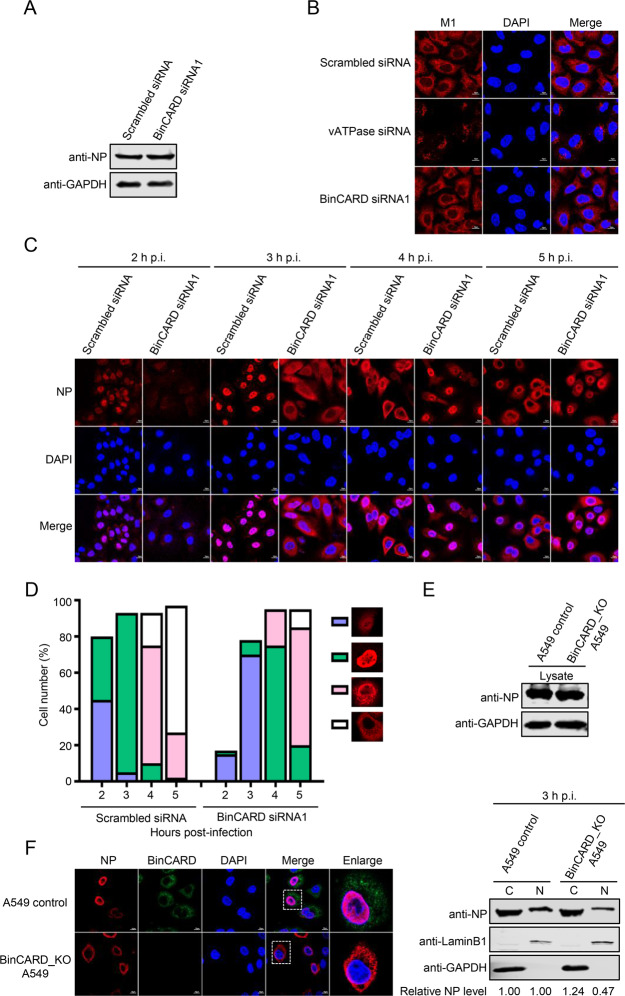
BinCARD1 positively regulates the nuclear import of the vRNP complex and newly synthesized NP. **A** The effect of BinCARD siRNA1 treatment on the internalization of IAV was analyzed. BinCARD siRNA1- or scrambled siRNA-treated A549 cells were infected with WSN (H1N1) virus (MOI = 5) on ice at 4 °C for 1 h, incubated at 37 °C for 30 min to allow viral internalization, and washed with ice-cold PBS (pH = 1.3) to remove uninternalized viral particles. Cell lysates were subjected to western blotting with a mouse anti-NP mAb to detect the amount of internalized virus particles. **B** The effect of BinCARD siRNA1 treatment on the uncoating process of IAV was analyzed. BinCARD siRNA1-, scrambled siRNA- or vATPase siRNA-treated A549 cells were treated with CHX to inhibit protein synthesis and were then infected with WSN (H1N1) (MOI = 50) virus. At 1.5 h p.i., the infected cells were stained with a rabbit anti-M1 pAb and Alexa Fluor 633 goat anti-rabbit IgG (H + L) (red) and visualized by confocal microscopy. **C** The effect of BinCARD siRNA1 treatment on the cellular localization of NP during IAV infection was determined by confocal microscopy. BinCARD siRNA1- or scrambled siRNA-treated A549 cells were infected with WSN (H1N1) (MOI = 5) virus. At 2, 3, 4 and 5 h p.i., the infected cells were fixed and stained with a mouse anti-NP mAb, followed by incubation with Alexa Fluor 633 goat anti-mouse IgG (H + L) (red). The nuclei were stained with DAPI. **D** Quantitative analysis of NP localization in virus-infected BinCARD siRNA1-treated cells. On the basis of the confocal microscopy images presented in **C**, the localization of NP (indicative of vRNP localization) upon the appearance of its nuclear localization was divided into the following categories: weak nuclear localization, strong nuclear localization, simultaneous localization at the boundary between the nucleus and the cytoplasm, or predominant cytoplasmic localization. The data shown are derived from 100 cells visualized by confocal microscopy with a 40X objective lens. **E** The effect of BinCARD1 knockout on the nuclear import of the vRNP complex was determined through a cell fractionation experiment. BinCARD_KO A549 cells and A549 control cells were infected with WSN (H1N1) (MOI = 5) virus. At 3 h p.i., the cells were separated into nuclear (N) and cytoplasmic (C) fractions. The proteins in each fraction were subjected to western blotting with a mouse anti-NP mAb. Densitometric analysis of the western blots was performed with ImageJ software. Lamin B1 and GAPDH were used as the loading controls for the nuclear and cytoplasmic fractions, respectively. **F** The effect of BinCARD1 knockout on the nuclear import of NP was assessed by confocal microscopy. BinCARD_KO A549 cells and A549 control cells were transfected with a vector to express WSNNP. At 20 h p.t., the cells were stained with a mouse anti-NP mAb and a rabbit anti-BinCARD pAb, incubated with Alexa Fluor 633 goat anti-mouse IgG (H + L) (red) and Alexa Fluor 488 goat anti-rabbit IgG (H + L) (green), and visualized by confocal microscopy

To define the specific stage of the IAV replication cycle in which BinCARD1 is engaged, we first examined whether BinCARD1 plays a role in viral internalization. BinCARD siRNA1- or scrambled siRNA-treated A549 cells were infected with WSN (H1N1) virus (MOI = 5) for 1 h on ice at 4 °C, and then, the temperature was changed to 37 °C for 30 min to allow viral internalization. After removing virions retained on the cell surface by washing with PBS (pH = 1.3), the amount of internalized viral NP protein was evaluated by western blotting. The amount of NP inside the BinCARD siRNA1-treated A549 cells was similar to that in the scrambled siRNA-treated cells (Fig. 2A), indicating that knocking down BinCARD1 expression had no adverse effect on the viral endocytic process. Next, we asked whether BinCARD1 affects the uncoating process of virus upon entry into cells. SiRNA-treated A549 cells were infected with a high dose of WSN (H1N1) virus (MOI = 50) in the presence of cycloheximide (CHX, 50 μg/mL) to exclude the influence of newly synthesized proteins. The cells were fixed at 1.5 h p.i., stained with an anti-M1 pAb to reveal the uncoating process, and visualized with confocal microscopy. We found that M1 was distributed as puncta in cells treated with siRNA against vATPase (the positive control), indicating that virions were not uncoated (Fig. 2B). In contrast, M1 was diffusely distributed in the cytoplasm of A549 cells treated with BinCARD siRNA1 or scrambled siRNA, indicating completion of the uncoating process; no difference in M1 distribution was observed between the cells treated with BinCARD siRNA1 or scrambled siRNA. Hence, knocking down BinCARD1 did not affect virion uncoating. Together, these results clearly indicate that BinCARD1 was neither involved in the internalization nor the uncoating process in the early stage of the IAV replication cycle.

To determine whether BinCARD1 modulates the nuclear import of the vRNP complex, we monitored the cellular localization of NP during the course of IAV infection in siRNA-treated A549 cells. A549 cells were treated with BinCARD siRNA1 or scrambled siRNA and infected with WSN (H1N1) virus (MOI = 5). At 2, 3, 4, and 5 h p.i., the cellular distribution of NP was visualized by confocal microscopy. NP clearly accumulated in the nucleus of approximately 35% and 88% of the scrambled siRNA-treated cells at 2 and 3 h p.i., respectively. However, the ratio of cells with NP accumulation in the nucleus of BinCARD siRNA1-treated cells was 2% and 8%, respectively, at the same timepoints. These results indicate that the vRNP complex is detained in the cytoplasm at 2 and 3 h p.i. in BinCARD siRNA1-treated cells. Due to the defect in the nuclear import of the vRNP complex, the replication cycle of IAV was delayed in the BinCARD siRNA1-treated cells, as indicated by the localization of NP at 4 and 5 h p.i. (Fig. 2C, D). The role played by BinCARD1 in the nuclear import of the vRNP complex after IAV infection was also examined in BinCARD_KO A549 cells. We found that the nuclear import of the vRNP complex was suppressed and that the overall viral replication cycle was delayed in the BinCARD_KO A549 cells compared with the A549 control cells (Supplementary Fig. 6A, B).

We next validated the inhibitory effect of BinCARD1 knockout on the nuclear import of the vRNP complex by performing a cell fractionation experiment. BinCARD_KO and control A549 cells were infected with WSN (H1N1) virus (MOI = 5). At 3 h p.i., the infected cells were lysed, and the cytoplasmic and nuclear fractions were separated and subjected to western blotting. The marker proteins GAPDH and LaminB1 were detected only in the cytoplasm and nucleus, respectively (Fig. 2E). Notably, the amount of NP detected in the nucleus of the BinCARD_KO A549 cells was much lower than that of the control cells (Fig. 2E). These results demonstrate that knocking out BinCARD1 expression suppressed the nuclear accumulation of the vRNP complex, thereby inhibiting viral life cycle progression.

We further investigated the effect of BinCARD1 on the nuclear import of NP protein. BinCARD_KO A549 cells and A549 control cells were transfected with a WSNNP-expressing construct, and the cellular localization of NP was visualized at 20 h p.t. NP accumulated in the nucleus of the A549 control cells but was predominantly retained in the cytoplasm of the BinCARD_KO A549 cells (Fig. 2F).

Collectively, these data demonstrate that BinCARD1 promoted the nuclear import of both the vRNP complex and newly synthesized NP.

### BinCARD1 increases the vRNP complex activity of IAV

**Fig. 3:**
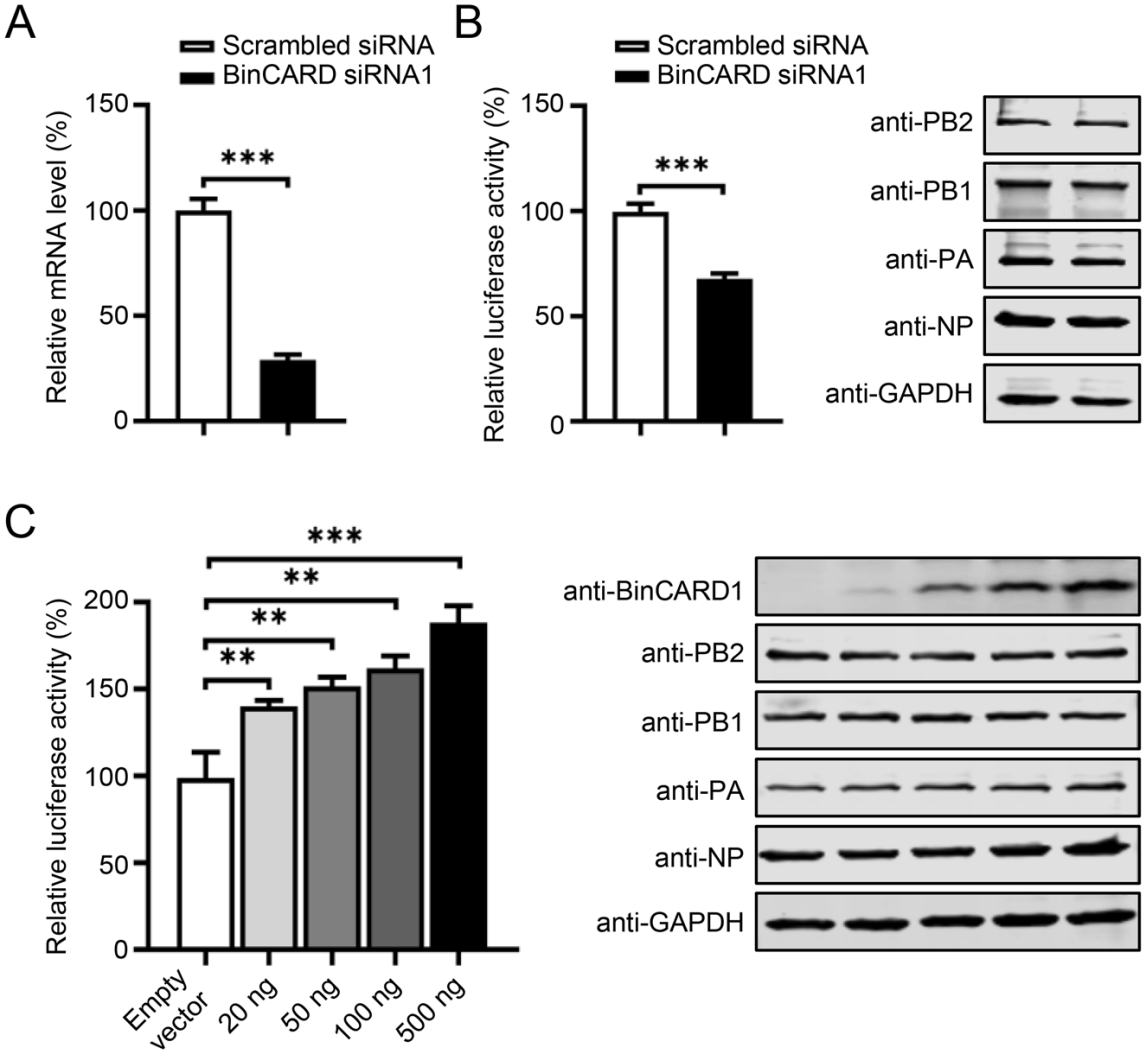
BinCARD1 increases the vRNP complex activity of IAV. **A** SiRNA knockdown of BinCARD in HEK293T cells. HEK293T cells were transfected with BinCARD siRNA1 or scrambled siRNA, and at 36 h p.t., the cells were subjected to RT‒qPCR analysis to determine the effect of BinCARD knockdown. ***P < 0.001. **B** The vRNP complex activity of IAV in BinCARD siRNA1-treated HEK293T cells. HEK293T cells treated with BinCARD siRNA1 or scrambled siRNA as described in **A** were further transfected with the set of plasmids for the evaluation of vRNP complex activity, including the four RNP protein expression constructs (PB2, PB1, PA, and NP) derived from WSN (H1N1) virus, pHH21-SC09NS F-Luc, and pRL-TK. Thirty-six hours later, a dual-luciferase assay was performed in which the relative firefly luciferase activity was normalized to the luciferase activity of the Renilla, the internal control. The expression of vRNP complex proteins was measured by western blotting with a mouse anti-PB2, PB1, PA, or NP mAb. ***P < 0.001. **C** The vRNP complex activity of IAV in HEK293T cells was increased with progressively increased BinCARD1 expression. HEK293T cells were transfected with the set of plasmids for the evaluation of vRNP complex activity as described in **B**, together with an empty vector or gradually increasing amounts of BinCARD1-expressing constructs. Thirty-six hours later, a dual-luciferase assay was performed as described in **B**. The expression of the BinCARD1 and vRNP complex proteins was measured by western blotting with a mouse anti-BinCARD1, PB2, PB1, PA, or NP mAb. **P < 0.01; ***P < 0.001. The data represent the mean ± SD of three independent experiments (**A**–**C**)

The vRNP complex is critical for the transcription and replication of the IAV genome [7]. Given the role played by BinCARD1 in the nuclear import of the vRNP complex, we speculated that BinCARD1 might increase vRNP complex activity. To test this hypothesis, we transfected HEK293T cells with BinCARD siRNA1 or scrambled siRNA. A RT‒qPCR analysis showed that BinCARD-specific siRNA treatment indeed downregulated the expression of BinCARD at 36 h p.t. (Fig. 3A). SiRNA-treated cells were then transfected with protein expression constructs for the PB2, PB1, PA, and NP of WSN (H1N1) virus, along with a reporter plasmid for the generation of a vRNA-like luciferase gene [20, 44, 45]. Thirty-six hours later, the luciferase activity in the cell lysates was measured to determine vRNP complex activity. We found that the vRNP activity was decreased by 33% in cells treated with BinCARD siRNA1 compared with cells treated with scrambled siRNA (Fig. 3B). The level of each vRNP complex protein remained unchanged between the two types of siRNA-treated cells, indicating that BinCARD1 did not exert an effect on vRNP complex activity by changing the expression levels of vRNP complex proteins. To confirm the result observed in siRNA-treated cells, the vRNP complex activity of WSN (H1N1) virus was assessed in the presence of exogenously expressed BinCARD1. We found that BinCARD1 overexpression led to dose-dependent increases in vRNP complex activity without affecting the expression level of any vRNP complex protein (Fig. 3C). These data demonstrate that BinCARD1 positively regulated the vRNP complex activity of IAV.

### BinCARD1 interacts with IAV NP

**Fig. 4:**
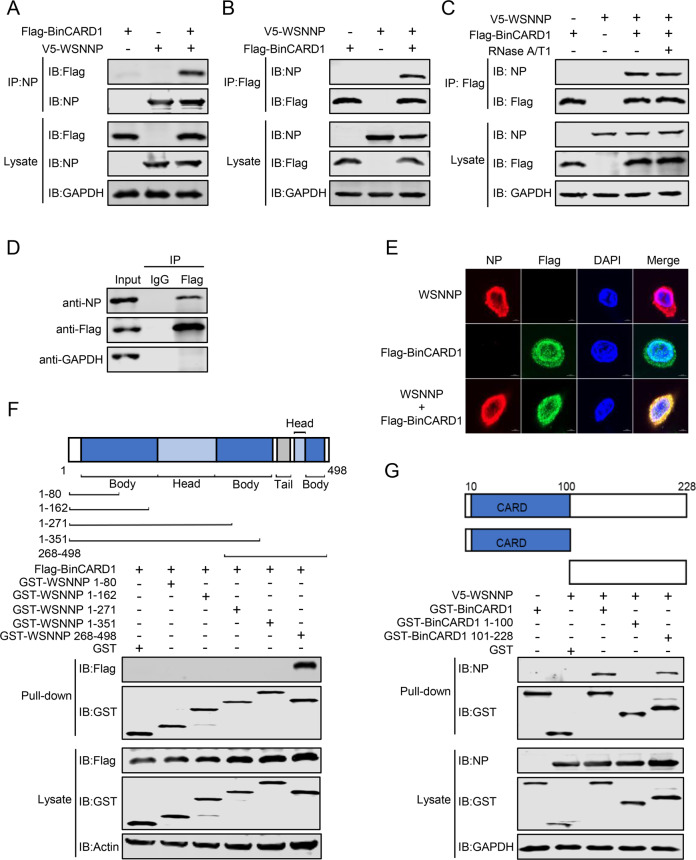
BinCARD1 interacts with NP. **A**, **B** A co-IP assay was performed to examine the interaction between NP and BinCARD1. HEK293T cells were transfected with plasmids expressing V5-WSNNP and Flag-BinCARD1 individually or in combination. At 36 h p.t., cell lysate proteins were immunoprecipitated with a mouse anti-NP mAb (**A**) or a mouse anti-Flag mAb (**B**) and subjected to western blotting with a rabbit anti-NP pAb or a rabbit anti-Flag pAb. **C** The BinCARD1-NP interaction did not rely on the RNA-binding activity of NP. HEK293T cells were transfected as described in **A**, **B**. Cell lysates treated with RNase A/T1 or left untreated were immunoprecipitated with a mouse anti-Flag mAb, and the proteins were subjected to western blotting with a rabbit anti-NP pAb or a rabbit anti-Flag pAb. **D** A co-IP assay was performed to examine the interaction between NP and BinCARD1 in virus-infected cells. HEK293T cells were transfected with vectors to express Flag-BinCARD1, and at 36 h p.t., the cells were infected with WSN (H1N1) virus (MOI = 5) for 12 h. Cell lysate proteins were immunoprecipitated with a mouse anti-Flag mAb and then subjected to western blotting with a rabbit anti-NP pAb or a rabbit anti-Flag pAb. **E** Colocalization of NP and BinCARD1 was detected by confocal microscopy. A549 cells were transfected with plasmids expressing WSNNP and Flag-BinCARD1 individually or in combination. At 20 h p.t., the cells were stained with a mouse anti-NP mAb and a rabbit anti-Flag pAb, incubated with Alexa Fluor 633 goat anti-mouse IgG (H + L) (red) and Alexa Fluor 488 goat anti-rabbit IgG (H + L) (green), and visualized by confocal microscopy. **F**, **G** Co-IP assays were performed to examine the interaction between truncation mutants of NP and BinCARD1. HEK293T cells were transfected with the indicated constructs. At 36 h p.t., cell lysates were pulled down with glutathione magnetic beads. The bound proteins were eluted and subjected to western blotting with a rabbit anti-NP pAb, a rabbit anti-Flag pAb, or  a rabbit anti-GST pAb

The nuclear import of the vRNP complex after IAV infection is mediated by the interaction between NP and IMP α isoforms. Since BinCARD1 positively regulated the nuclear import of the vRNP complex and NP, we investigated whether BinCARD1 binds to NP by performing co-IP experiments. HEK293T cells were transfected with V5-tagged WSNNP and Flag-tagged BinCARD1 individually or in combination, and cell lysate proteins were immunoprecipitated with a mouse anti-NP mAb or anti-Flag mAb, and then, western blotting with a rabbit anti-NP pAb or anti-Flag pAb was performed. A clear interaction between NP and BinCARD1 was observed (Fig. 4A, B). Given that the RNA-binding activity of NP is important for its function in the life cycle of IAV, we performed a co-IP experiment with cell lysates that had been first treated with RNase A/T1 to determine whether the interaction between NP and BinCARD1 depends on the RNA-binding activity of NP. We found that the interaction between BinCARD1 and NP was not affected by the removal of the RNA components in the lysates of the transfected cells (Fig. 4C), indicating that the BinCARD1 and NP interaction did not rely on the RNA-binding activity of NP. We also examined the interaction between NP and BinCARD1 in infected cells. HEK293T cells transfected with plasmids expressing Flag-BinCARD1 were infected with WSN (H1N1) virus and then subjected to co-IP. We found that BinCARD1 interacted with NP during the course of IAV infection (Fig. 4D). Furthermore, we examined the interaction between NP and BinCARD1 via confocal microscopy and found that WSNNP and Flag-BinCARD1 colocalized in the nucleus and cytoplasm of transiently transfected A549 cells (Fig. 4E).

Next, we investigated the key regions within NP and BinCARD1 that are critical for their interaction. Five previously generated NP truncation constructs were used to express peptides with only the amino acid regions of 1–80, 1–162, 1–271, 1–351, and 268–498 [20]. Similarly, we generated two truncation constructs of BinCARD1, one expressed the N-terminal 1–100 amino acids and the other expressing the C-terminal 101–228 amino acids. A GST tag was fused to each truncated NP and BinCARD1 construct at the N-terminus. We then examined the interaction between BinCARD1 and GST-NP truncation mutants as well as the interaction between NP and GST-BinCARD1 truncation mutants by performing a GST pull-down assay. The C-terminal 352–498 amino acid region of NP was found to mediate the interaction of NP with BinCARD1 (Fig. 4F), and the C-terminal 101–228 amino acid region of BinCARD1 was found to be critical for BinCARD1 binding with NP (Fig. 4G).

### BinCARD1 facilitates the binding of NP with IMP α7

**Fig. 5:**
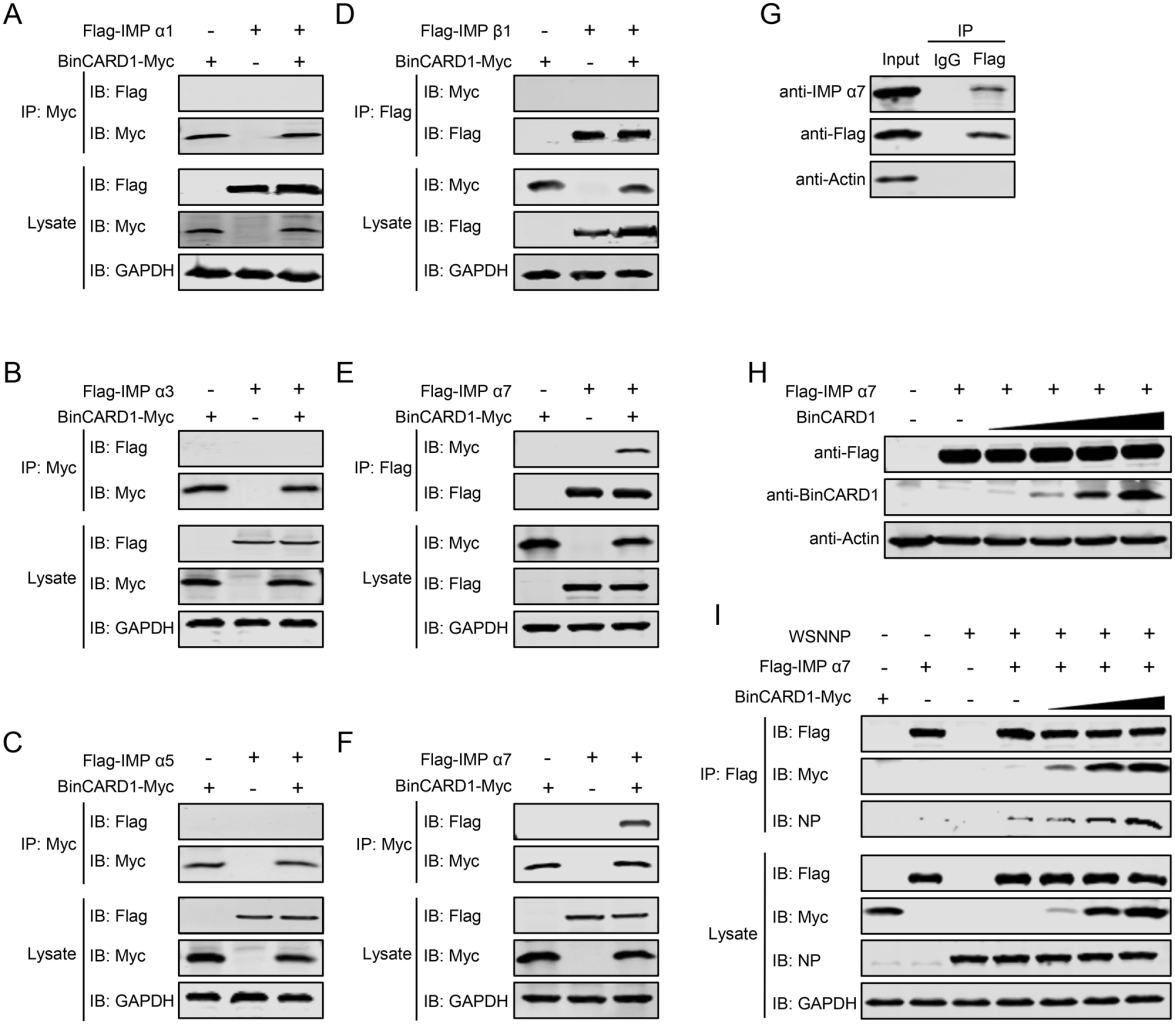
BinCARD1 facilitates the binding of NP with IMP α7. **A**–**D** Co-IP assays were performed to examine the interaction between BinCARD1 and IMP α1, α3, α5, and β1. HEK293T cells were transfected with plasmids expressing Myc-tagged BinCARD1 and Flag-tagged IMP α1 (**A**), α3 (**B**), α5 (**C**), or β1 (**D**) individually or in combination. At 36 h p.t., cell lysate proteins were immunoprecipitated with a mouse anti-Myc mAb or a mouse anti-Flag mAb and subjected to western blotting with a rabbit anti-Myc pAb or a rabbit anti-Flag pAb. **E**, **F** A co-IP assay was performed to examine the interaction between BinCARD1 and IMP α7. HEK293T cells were transfected with plasmids expressing Myc-tagged BinCARD1 and Flag-tagged IMP α7 individually or in combination. At 36 h p.t., cell lysate proteins were immunoprecipitated with a mouse anti-Flag mAb (**E**) or a mouse anti-Myc mAb (**F**) and subjected to western blotting with a rabbit anti-Myc pAb or a rabbit anti-Flag pAb. **G** A co-IP assay was performed to examine the interaction between BinCARD1 and endogenous IMP α7 during IAV infection. HEK293T cells were transfected with vectors to express Flag-tagged BinCARD1, and at 36 h p.t., the cells were infected with WSN (H1N1) virus (MOI = 5) for 12 h. Cell lysate proteins were immunoprecipitated with a mouse anti-Flag mAb and subjected to western blotting with a rabbit anti-IMP α7 pAb or a rabbit anti-Flag pAb. **H** Western blotting analysis to determine the effect of gradually increasing BinCARD1 expression on the expression of IMP α7. HEK293T cells were transfected with vectors to express Flag-tagged IMP α7 and gradually increasing levels of BinCARD1. At 36 h p.t., cell lysate proteins were western blotted with a rabbit anti-Flag pAb and a mouse anti-BinCARD1 mAb. **I** Co-IP assays were performed to examine the relationship among NP, IMP α7, and BinCARD1. HEK293T cells were transfected with plasmids expressing WSNNP, Flag-tagged IMP α7, and gradually increasing amounts of Myc-tagged BinCARD1 individually or in combination. At 36 h p.t., cell lysate proteins were immunoprecipitated with a mouse anti-Flag mAb and subjected to western blotting with a rabbit anti-NP pAb, a rabbit anti-Flag pAb, or a rabbit anti-Myc pAb

It had previously been reported that the nuclear import of NP was mediated by its association with IMP α1, α3, α5, or α7 and the subsequent binding of IMP β1 [16, 17]. Given that BinCARD1 interacts with NP and facilitates the nuclear import of the vRNP complex and newly synthesized NP, we hypothesized that BinCARD1 might affect the interaction between NP and IMP α isoforms or the subsequent recruitment of IMP β1. To test this hypothesis, we determined whether BinCARD1 interacts with IMP α isoforms or IMP β1 by performing co-IP assays. HEK293T cells were transfected with plasmids expressing Myc-tagged BinCARD1 and Flag-tagged IMP α1, α3, α5, α7, or β1 individually or in combination. Cell lysate proteins were immunoprecipitated with a mouse anti-Myc or anti-Flag mAb, and then, western blotting was performed with a rabbit anti-Myc or anti-Flag pAb. The data revealed that BinCARD1 did not interact with IMP α1, α3, α5, or β1 (Fig. 5A–D). In contrast, we discovered that BinCARD1 interacts with IMP α7 (Fig. 5E, F). The interaction between BinCARD1 and IMP α7 was also observed during the course of IAV infection, as determined with a co-IP assay performed with HEK293T cells transfected with plasmids expressing Flag-BinCARD1 and subsequently infected with WSN (H1N1) virus (Fig. 5G).

We next investigated the biological effect of the interaction between BinCARD1 and IMP α7. We found that increasing the amount of BinCARD1 in HEK293T cells exerted no effect on the expression level of IMP α7 (Fig. 5H). We further examined whether the interaction between BinCARD1 and IMP α7 promotes complex formation between NP and IMP α7, thus facilitating the nuclear import of NP through the classical nuclear import pathway. HEK293T cells were transfected with plasmids expressing WSNNP and Flag-tagged IMP α7, together with gradually increasing amounts of the Myc-tagged BinCARD1 construct. The co-IP assay showed that the amount of NP that coimmunoprecipitated with IMP α7 was significantly increased in a dose-dependent manner when BinCARD1 was coexpressed (Fig. 5I). This result indicates that the presence of BinCARD1 promoted the formation of a complex between NP and IMP α7, thereby facilitating the nuclear import of the vRNP complex.

### BinCARD1 activates RIG-I innate immune signaling by promoting the K63-linked ubiquitination of TRAF3

**Fig. 6:**
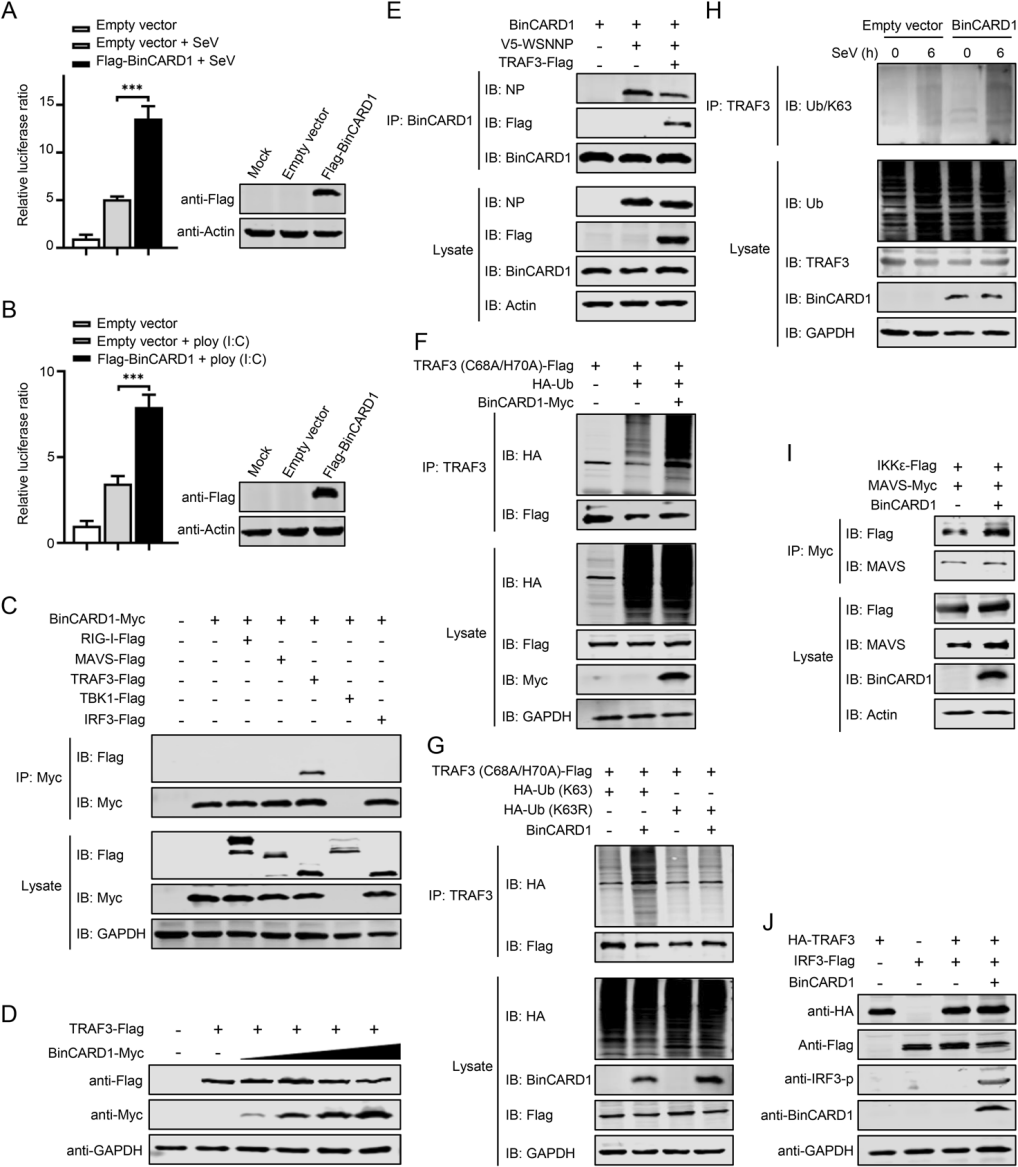
BinCARD1 activates RIG-I signaling by promoting the K63-linked ubiquitination of TRAF3. **A**, **B** An ISRE luciferase reporter assay was performed to determine the effect of BinCARD1 on the activation of RIG-I signaling. HEK293T cells were transfected with an ISRE-Luc reporter plasmid, a pRL-TK control plasmid, and a Flag-BinCARD1-expressing plasmid or an empty pCAGGS vector. At 36 h p.t., the cells were left untreated or stimulated with SeV (**A**) or poly (I:C) (**B**) for 12 h. The overexpression of BinCARD1 was confirmed by western blotting with a rabbit anti-Flag pAb. The luciferase activity of the cell lysates was analyzed with a dual-luciferase reporter assay in which firefly luciferase activity was normalized to the luciferase activity of Renilla, which had been coexpressed. The fold-change in the firefly luciferase activity of BinCARD1-overexpressing cells compared with cells transfected with empty vector is shown. ***P < 0.001. **C** Co-IP assays were performed to examine the interaction between BinCARD1 and RIG-I signaling pathway proteins. HEK293T cells were transfected with plasmids expressing Myc-tagged BinCARD1 and Flag-tagged RIG-I, MAVS, TRAF3, TBK1, or IRF3. At 36 h p.t., cell lysate proteins were immunoprecipitated with a mouse anti-Myc mAb and subjected to western blotting with a rabbit anti-Flag pAb or a rabbit anti-Myc pAb. **D** HEK293T cells were transfected with plasmids expressing Flag-tagged TRAF3 and gradually increasing amounts of Myc-tagged BinCARD1. At 36 h p.t., cell lysate proteins were subjected to western blotting with a rabbit anti-Flag pAb or a rabbit anti-Myc pAb. **E** TRAF3 impaired the binding between BinCARD1 and IAV NP. HEK293T cells were transfected with combinations of plasmids expressing BinCARD1, V5-WSNNP, and TRAF3-Flag. At 36 h p.t., cell lysate proteins were immunoprecipitated with a mouse anti-BinCARD1 mAb, and the bound proteins were eluted and subjected to western blotting with a mouse anti-BinCARD1 mAb, a rabbit anti-Flag pAb, or a rabbit anti-NP pAb. **F** A co-IP assay was performed to assess TRAF3 ubiquitination. HEK293T cells were transfected with plasmids expressing Flag-tagged TRAF3 (C68A/H70A) and HA-tagged ubiquitin, with or without Myc-tagged BinCARD1. At 36 h p.t., cell lysate proteins were immunoprecipitated with a mouse anti-TRAF3 mAb and then western blotted with a rabbit anti-Flag pAb or a rabbit anti-HA mAb. **G** A co-IP assay was performed to determine the type of TRAF3 ubiquitination linkage mediated by BinCARD1. HEK293T cells were transfected with plasmids expressing Flag-tagged TRAF3 (C68A/H70A) and HA-tagged ubiquitin (K63) or ubiquitin (K63R), with or without BinCARD1. At 36 h p.t., cell lysate proteins were immunoprecipitated with a mouse anti-TRAF3 mAb and subjected to western blotting with a rabbit anti-Flag pAb or a rabbit anti-HA mAb. **H** K63-linked polyubiquitination of TRAF3 was induced by BinCARD1 in infected cells. RAW264.7 cells were transfected with an empty vector or a BinCARD1-expressing plasmid, and at 48 h p.t., the cells were infected with SeV. At 0 and 6 h p.i., cell lysate proteins were immunoprecipitated with a mouse anti-TRAF3 mAb and subjected to western blotting with a rabbit anti-ubiquitin (linkage-specific K63) mAb, a mouse anti-ubiquitin mAb, a mouse anti-TRAF3 mAb, or a mouse anti-BinCARD1 mAb. **I** A co-IP assay was performed to determine the effect of BinCARD1 on the interaction between MAVS and IKKε. HEK293T cells were transfected with plasmids expressing Flag-tagged IKKε and Myc-tagged MAVS, with or without BinCARD1. At 36 h p.t., cell lysate proteins were immunoprecipitated with a mouse anti-Myc mAb and subjected to western blotting with a rabbit anti-Flag pAb or a rabbit anti-MAVS mAb. **J** The effect of BinCARD1 on the phosphorylation of IRF3 was assessed. HEK293T cells were transfected with plasmids expressing HA-tagged TRAF3, Flag-tagged IRF3, and BinCARD1 individually or in various combinations. At 36 h p.t., cell lysate proteins were subjected to western blotting with a rabbit anti-HA mAb, a rabbit anti-Flag pAb, a rabbit anti-IRF3-p mAb, or a mouse anti-BinCARD1 mAb

When host factors promote the replication of IAV, it is inevitable that mechanisms of host antagonism are activated. Type I IFN plays an important role in host antiviral immunity. Given that BinCARD2 can induce MAVS oligomerization to activate the RIG-I innate immune pathway [39], we hypothesized that BinCARD1 may also play a role in RIG-I innate immune signaling. To test this hypothesis, HEK293T cells were transfected with an ISRE luciferase reporter gene and a BinCARD1 expression construct or an empty vector and were then left untreated or treated with inducers of type I IFN activity [i.e., SeV or poly (I:C)]. By measuring the luciferase activity, we found that cells with overexpressed BinCARD1 showed dramatically enhanced SeV- or poly (I:C)-induced expression of the ISRE luciferase reporter gene compared with cells that did not overexpress BinCARD1 (Fig. 6A, B). Consistent with these data, BinCARD1 knockdown in A549 cells, realized through BinCARD siRNA1 treatment, led to a reduction in IRF3 phosphorylation after SeV stimulation compared with the level in scrambled siRNA-treated cells (Supplementary Fig. 7A). In addition, HEK293T cells were transfected with plasmid expressing  BinCARD1 or an empty vector and then stimulated with SeV or poly (I:C) for 6 h. A RT‒qPCR analysis showed that the expression levels of ISG15 and OAS1, two of the most upregulated type I IFN responsive genes, were much higher in the BinCARD1-overexpressing cells than in the control cells (Supplementary Fig. 7B–E). These data indicate that BinCARD1 was involved in the activation of RIG-I signaling.

To investigate the elements of the RIG-I pathway affected by BinCARD1 function, we performed co-IP experiments with HEK293T cells to examine whether BinCARD1 associates with key adaptors in the RIG-I pathway. BinCARD1 clearly interacted with TRAF3 but not RIG-I, MAVS, TBK1, or IRF3 (Fig. 6C). We found that increasing amounts of BinCARD1 in HEK293T cells exerted no effect on the expression level of TRAF3 (Fig. 6D). Interestingly, we did not detect BinCARD1 in the lysates of cells in which Myc-tagged BinCARD1 and Flag-tagged TBK1 were coexpressed (Fig. 6C). We therefore speculated that BinCARD1 might be degraded by TBK1.

Because BinCARD1 interacts with both IAV NP and TRAF3, we explored whether TRAF3 competes with NP for binding to BinCARD1. A co-IP assay showed that the amount of NP coimmunoprecipitated with BinCARD1 was dramatically reduced when TRAF3 was also expressed (Fig. 6E), indicating that the binding between NP and BinCARD1 was inhibited by the presence of TRAF3.

In the RIG-I pathway, K63-linked ubiquitination of TRAF3 is required for the induction of type I IFNs [46, 47]. We therefore determined whether BinCARD1 activates TRAF3. A TRAF3 mutant deficient in E3 ubiquitin ligase activity (C68A/H70A) was assessed because this mutant is unable to mediate the ubiquitination of substrates, including itself through autoubiquitination [48]. BinCARD1-mediated polyubiquitination of TRAF3 was readily detected in HEK293T cells expressing Flag-tagged TRAF3 (C68A/H70A), HA-tagged ubiquitin, and Myc-tagged BinCARD1 (Fig. 6F). CARD domain-containing proteins can bind unanchored K63 ubiquitin chains, thereby activating target proteins [49, 50]. Therefore, to determine whether the expression of BinCARD1 leads to the K63-linked polyubiquitination of TRAF3, we cotransfected HEK293T cells with different combinations of plasmids to express Flag-tagged TRAF3 (C68A/H70A), BinCARD1, and ubiquitin mutant in which either all lysine residues except K63 were replaced with arginine residues (Ub-K63) or only the K63 residue was replaced with an arginine residue (Ub-K63R). Co-IP analyses showed that BinCARD1 promoted the ubiquitination of TRAF3 (C68A/H70A) in the presence of Ub-K63 but not Ub-K63R (Fig. 6G), indicating that BinCARD1 promoted the K63-linked ubiquitination of TRAF3. Consistent with these data, the SeV-induced K63-linked polyubiquitination of endogenous TRAF3 was clearly enhanced in BinCARD1-overexpressing RAW264.7 cells compared with control cells (Fig. 6H). We further examined the effect of BinCARD1-mediated K63-linked ubiquitination of TRAF3 on downstream signaling in the RIG-I pathway. The interaction between MAVS and IKKε was markedly increased when BinCARD1 was expressed (Fig. 6I). We also measured the phosphorylation rate of IRF3 in the absence or presence of BinCARD1 overexpression. HEK293T cells were transfected with vectors to express TRAF3 and IRF3 in the presence or absence of BinCARD1. We found that when coexpressed with BinCARD1, IRF3 was significantly phosphorylated (Fig. 6J). These results indicate that the K63-linked polyubiquitination of TRAF3, catalyzed by BinCARD1, was important for the recruitment of the TBK1-IKKε kinase complex and activation of RIG-I signaling.

### TBK1 mediates BinCARD1 degradation through the autophagy pathway

**Fig. 7:**
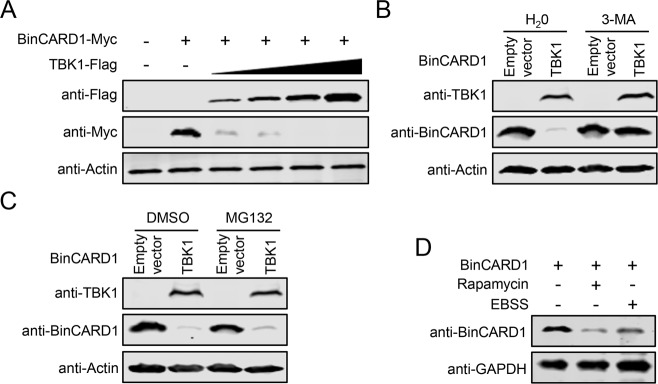
TBK1 mediates the degradation of BinCARD1 through the autophagy pathway. **A** TBK1-mediated degradation of BinCARD1 was analyzed by western blotting. HEK293T cells were transfected with plasmids expressing Myc-tagged BinCARD1 and gradually increasing amounts of Flag-tagged TBK1 individually or in combination. At 36 h p.t., cell lysate proteins were subjected to western blotting with a rabbit anti-Flag pAb or a rabbit anti-Myc pAb. **B**, **C** Western blotting was performed to identify the pathway through which TBK1 degrades BinCARD1. HEK293T cells were transfected with plasmids expressing BinCARD1, and at a 12-hour interval, the cells were transfected with an empty vector or a TBK1-expressing plasmid. Twenty-four hours later, the cells were treated for 8 h with H_2_O or 3-MA (**B**) and DMSO or MG132 (**C**). Cell lysate proteins were subjected to western blotting with a mouse anti-BinCARD1 mAb or a rabbit anti-TBK1 mAb. **D** The effect of autophagy inducers on the level of BinCARD1 was assessed. HEK293T cells were transfected with plasmids expressing BinCARD1. At 24 h p.t., cells were treated with rapamycin for 24 h or EBSS for 2 h. Cell lysate proteins were subjected to western blotting with a mouse anti-BinCARD1 mAb

TBK1 is an important regulator of not only the innate immune system but also autophagy [36, 51, 52]. It has also been documented that TBK1 functions as an E3 ubiquitin ligase to degrade target proteins [53]. When coexpressed with TBK1, BinCARD1 was undetectable (Fig. 6C). In addition, with increases in the abundance of exogenous TBK1-Flag, the amount of Myc-BinCARD1 gradually decreased (Fig. 7A). These results prompted us to hypothesize that BinCARD1 might be degraded by TBK1 via the ubiquitin proteasome pathway or autophagy pathway. Therefore, we used the proteasome inhibitor MG132 and the autophagy inhibitors 3-MA and Baf A1 to identify the pathway through which BinCARD1 is degraded by TBK1. We found that the degradation of BinCARD1 was abolished in the presence of 3-MA or Baf A1 but not MG132 (Fig. 7B, C, Supplementary Fig. 8), indicating that the autophagy activation was required for the degradation of BinCARD1 by TBK1. To confirm this result, we treated HEK293T cells that had been transfected with vectors to overexpress BinCARD1 with the autophagy inducers rapamycin and EBSS. We found that the addition of these autophagy inducers dramatically reduced the level of BinCARD1 (Fig. 7D), indicating that the degradation of BinCARD1 was indeed mediated by TBK1 via the autophagy system. To map the region of TBK1 involved in the degradation of BinCARD1, we generated four TBK1 truncation constructs tagged with Flag at the C-terminus and assessed their effects on the degradation of BinCARD1 in HEK293T cells. BinCARD1 was degraded in the presence of wild-type (WT) TBK1 and the truncated mutants TBK1-KD + ULD and TBK1-ULD + CC (Supplementary Fig. 9), indicating that the ULD domain in TBK1 was important for the degradation of BinCARD1.

### TBK1 promotes the autophagic degradation of BinCARD1 by activating p62 phosphorylation

**Fig. 8:**
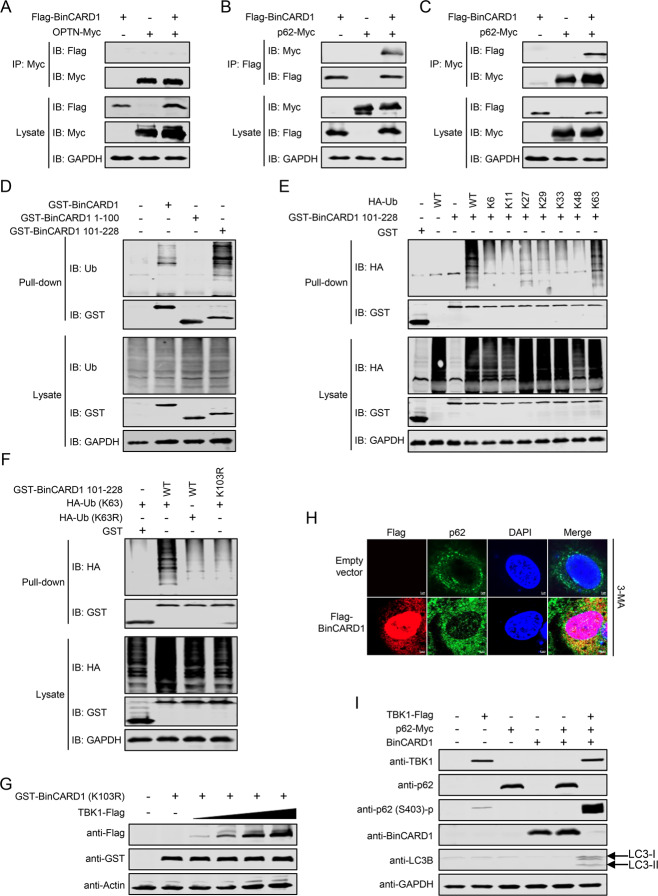
TBK1 promotes the autophagic degradation of BinCARD1 by activating p62 phosphorylation. **A** A co-IP assay was performed to examine the interaction between BinCARD1 and OPTN. HEK293T cells were transfected with plasmids expressing Flag-tagged BinCARD1 and Myc-tagged OPTN individually or in combination. At 36 h p.t., cell lysate proteins were immunoprecipitated with a mouse anti-Myc mAb and subjected to western blotting with a rabbit anti-Myc pAb or a rabbit anti-Flag pAb. **B**, **C** A co-IP assay was performed to examine the interaction between BinCARD1 and p62. HEK293T cells were transfected with plasmids expressing Flag-tagged BinCARD1 and Myc-tagged p62 individually or in combination. At 36 h p.t., cell lysate proteins were immunoprecipitated with a mouse anti-Flag mAb (**B**) or a mouse anti-Myc mAb (**C**) and subjected to western blotting with a rabbit anti-Flag pAb or a rabbit anti-Myc pAb. **D** GST pull-down assays were performed to examine the endogenous ubiquitination of full-length and truncation mutants of BinCARD1. HEK293T cells were transfected with plasmids expressing GST-tagged BinCARD1, BinCARD1 1–100, or BinCARD1 101–228. Thirty-six hours later, cell lysate proteins were pulled down with glutathione magnetic beads, and the bound proteins were eluted and subjected to western blotting with a mouse anti-ubiquitin mAb or a rabbit anti-GST pAb to determine the presence of endogenous ubiquitin and BinCARD1, respectively. **E** A GST pull-down assay was performed to examine the type of ubiquitination linkage on BinCARD1 101–228. HEK293T cells were transfected with plasmids expressing the GST-tagged BinCARD1 101–228 mutant and HA-tagged WT ubiquitin or the indicated ubiquitin mutants. At 36 h p.t., cell lysate proteins were pulled down with glutathione magnetic beads, and the bound proteins were eluted and subjected to western blotting with a rabbit anti-HA mAb or a rabbit anti-GST pAb. **F** The ubiquitination of the K103R mutant of BinCARD1 101–228 in cells expressing ubiquitin mutants was analyzed. HEK293T cells were transfected with plasmids expressing WT or the K103R mutant of GST-tagged BinCARD1 101–228 and HA-tagged ubiquitin (K63) or ubiquitin (K63R). At 36 h p.t., cell lysate proteins were pulled down with glutathione magnetic beads, and the bound proteins were eluted and subjected to western blotting with a rabbit anti-HA mAb or a rabbit anti-GST pAb. **G** The effect of TBK1 on the stability of the BinCARD1 K103R ubiquitination mutant was evaluated. HEK293T cells were transfected with plasmids expressing the GST-tagged BinCARD1 K103R mutant and gradually increasing amounts of Flag-tagged TBK1 individually or in combination. At 36 h p.t., cell lysates were subjected to western blotting with a rabbit anti-Flag pAb or a mouse anti-GST mAb. **H** Confocal microscopy was performed to visualize the formation of cytoplasmic p62 puncta. A549 cells were transfected with Flag-BinCARD1-expressing plasmids or an empty pCAGGS vector, and at 24 h p.t., the cells were treated with the autophagy inhibitor 3-MA for 8 h. The cells were fixed and stained with a rabbit anti-Flag pAb and a mouse anti-p62 mAb and then incubated with Alexa Fluor 633 goat anti-rabbit IgG (H + L) (red) and Alexa Fluor 488 goat anti-mouse IgG (H + L) (green). The nuclei were stained with DAPI. **I** The TBK1-p62 axis mediated the autophagic degradation of BinCARD1. HEK293T cells were transfected with plasmids expressing Flag-tagged TBK1, Myc-tagged p62, and BinCARD1 individually or in combination. At 36 h p.t., cell lysates were subjected to western blotting with a rabbit anti-TBK1 mAb, a mouse anti-p62 mAb, a rabbit anti-phospho-SQSTM1/p62 (Ser403) mAb, a mouse anti-BinCARD1 mAb, or a rabbit anti-LC3B pAb

TBK1 can regulate autophagy by phosphorylating the autophagy receptors OPTN and p62 [54, 55]. We first asked whether BinCARD1 associates with OPTN or p62. Co-IP experiments in HEK293T cells showed that BinCARD1 did not interact with OPTN (Fig. 8A) but clearly interacted with p62 (Fig. 8B, C). Since ubiquitination is necessary for autophagic degradation of target proteins, we next examined whether overexpressed BinCARD1 is modified by endogenous ubiquitin chains. GST pull-down and western blot assays showed that BinCARD1 undergoes ubiquitination at the C-terminal 101–228 region but not the N-terminal 1–100 region (Fig. 8D). To identify the type of BinCARD1 polyubiquitination linkage, we expressed GST-BinCARD1 101–228 in HEK293T cells, together with HA-tagged WT ubiquitin (WT Ub) or Ub mutants in which all but one internal lysine residues were mutated. Coexpression of either WT Ub or the Ub-K63 mutant led to efficient BinCARD1 polyubiquitination, whereas coexpression of other Ub mutants did not exert this effect (Fig. 8E), indicating that BinCARD1 was ubiquitinated via the K63 linkage. There is only one lysine residue, located at position 103, within the 101–228 region of BinCARD1. By generating a GST-BinCARD1 101–228 mutant bearing a K103R mutation, we found that the K63-linked ubiquitination of BinCARD1 101–228 was abolished (Fig. 8F), confirming that the ubiquitination of BinCARD1 occurred at the K103 residue. Furthermore, HEK293T cells were cotransfected to express the GST-tagged BinCARD1 K103R mutant and Flag-tagged TBK1. We found that the amount of GST-tagged BinCARD1 K103R mutant remained stable in the presence of gradually increasing amounts of coexpressed Flag-tagged TBK1 (Fig. 8G). These results demonstrate that the ubiquitination of BinCARD1 was required for its autophagic degradation mediated by TBK1.

Previous studies have shown that TBK1 can phosphorylate the S403 site of p62 to activate p62 and induce p62 binding to target proteins, thereby promoting autophagy [35]. We reasoned that BinCARD1 is also likely degraded through this pathway. To test this idea, A549 cells were transfected with a Flag-BinCARD1-expressing plasmid or an empty pCAGGS vector. At 24 h p.t., the cells were treated with the autophagy inhibitor 3-MA for 8 h. A confocal microscopy analysis showed that in the presence of 3-MA, more endogenous p62 puncta were observed in the cytoplasm of the BinCARD1-overexpressing A549 cells than in the control cells (Fig. 8H), indicating that p62 played an important role in the active autophagic degradation of BinCARD1. To further validate the importance of the TBK1-p62 axis in the autophagic degradation of BinCARD1, HEK293T cells were transfected with plasmids expressing Flag-tagged TBK1, Myc-tagged p62, and BinCARD1 individually or in combination. At 36 h p.t., cell lysates were subjected to western blotting. We found that endogenous phosphorylation of p62 at site 403 was detectable when TBK1 was overexpressed. Notably, in comparison to cells coexpressing only p62 and BinCARD1, coexpression of TBK1, p62, and BinCARD1 led to a dramatic increase in phosphorylation of p62, induction of LC-3II, a marker of autophagy initiation, and degradation of BinCARD1 (Fig. 8I). These results demonstrate that TBK1-mediated phosphorylation of p62 promoted the autophagic degradation of BinCARD1.

## Discussion

During the life cycle of IAV, after viral fusion and uncoating, the vRNP complex is released into the cytoplasm, and the active cellular transport machinery delivers the vRNP complex to the nucleus [5]. Within the nucleus, the primary transcription catalyzed by the vRNP complex leads to the generation of mRNA, which is exported to ribosomes, where progeny viral proteins, including PB2, PB1, PA, and NP, are synthesized. These newly synthesized vRNP complex proteins re-enter the nucleus, either as monomers, in the cases of PB2 and NP, or as PB1-PA dimers, to complete the transcription and replication of the viral genome [56, 57]. During the nuclear import of the vRNP complex and newly synthesized NP, IMP α isoforms recognize the NLSs of NP and recruit IMP β to facilitate NP and vRNP complex crossing the nuclear pore complex for translocation into the nucleus of infected cells [11, 13]. Hence, any host factor that affects the interaction of NP with the classical nuclear import pathway influences the nuclear import of the vRNP complex and NP and, ultimately, the replication of IAV. To date, several host proteins have been reported to facilitate or interfere with the interaction between NP and the classical nuclear import pathway. For example, PLSCR1 forms a trimeric complex with NP and IMP α, blocking the recruitment of IMP β [20]; MOV10 inhibits the association between NP and IMP α [17]; and eEF1D interferes with the interaction between NP and IMP α5 [22]. In contrast, Hsp40/DnaJB1 promotes the binding of NP to IMP α [21]. In the present study, we identified BinCARD1 as an important host factor in the replication of IAV. Knocking down or knocking out BinCARD1 expression decreased the replication of IAV, whereas overexpression of BinCARD1 promoted viral growth. In contrast to host factors that function in the steps of internalization or uncoating (e.g., FFAR2, IGDCC4, HDAC6, and EPS8) [45, 58–60], BinCARD1 does not appear to affect these early steps of IAV entry. Instead, we found that downregulation of BinCARD1 expression suppressed the nuclear import of the vRNP complex and NP, whereas overexpression of BinCARD1 exerted an enhancing effect. Consistent with these data, the expression of BinCARD1 was found to be important for the vRNP complex activity of IAV. Mechanistically, BinCARD1 interacts with both NP and IMP α7 and facilitates the complex formation between NP and IMP α7, thereby promoting the nuclear import of the vRNP complex and NP.

The innate immune system is the first line of host defense against infection, which is essential for the initial detection and recognition of invading pathogens, activation of acute antimicrobial responses, and subsequent activation of adaptive immunity. When pathogens invade a host, pattern recognition receptors (PRRs) specifically recognize pathogen-associated molecular patterns (PAMPs). A number of PRRs have been identified, and their functions have been extensively studied; well-characterized PRRs include the cytoplasmic viral sensor RIG-I [24, 41]. During IAV infection, RIG-I senses 5’-ppp in the termini of viral RNAs, leading to the activation of innate immune signaling cascades and the secretion of type I IFNs. TRAF3 is an important adaptor in the RIG-I signaling pathway [61]. During viral infection, K63-linked polyubiquitination of TRAF3 recruits TBK1-IKKε, and IRF3 is then activated to trigger type I IFN production [30, 62]. Recently, it has been revealed that the CARD domain plays a role not only in CARD-CARD interactions but also in ubiquitination, in which unanchored free K63-linked ubiquitin chains bind to CARD and regulate CARD protein activation [49, 50]. K63-linked polyubiquitin chains play an important role in protein complex assembly and function [63, 64]. In this study, we found that the interaction between BinCARD1 and TRAF3 led to K63-linked polyubiquitination and activation of TRAF3, followed by the recruitment of downstream TBK1-IKKε and phosphorylation of IRF3, thereby promoting the activation of the RIG-I signaling pathway.

TBK1 is a ubiquitous member of the IKK family [65]. It was initially identified as a kinase critical for the expression of type I IFNs and is considered a master regulator of innate immunity signaling pathways [32, 66–69]. Increasing evidence suggests that, in addition to being involved in innate immunity, TBK1 is involved in autophagy [36, 70]. In the present study, we found that TBK1 degraded BinCARD1 when both proteins were exogenously coexpressed. Using autophagy inhibitors or inducers, we found that the TBK1-mediated degradation of BinCARD1 was dependent on the autophagy pathway. TBK1 induced autophagy by phosphorylating autophagy receptors, namely, p62 and OPTN [36, 70]. Activated autophagy receptors bind to proteins that subsequently undergo ubiquitination and interact with the autophagosomal protein LC3, which delivers the ubiquitinated proteins to an autophagosome for degradation, thus completing the process of autophagy [35, 71, 72]. We found that BinCARD1 interacted with p62 but not OPTN, indicating that the autophagic degradation of BinCARD1 was mediated by p62. In contrast to K48-linked polyubiquitin chains, which are canonical signals on substrate proteins that target them for proteasomal degradation, K63-ubiquitinated proteins are degraded in lysosomes through the selective autophagy pathway [73]. We confirmed that BinCARD1 was polyubiquitinated at position 103 via the K63 linkage, which led to the autophagic degradation of BinCARD1, and further revealed that the BinCARD1 K103R mutant remained stable when coexpressed with TBK1. Acting as a key adaptor in the autophagic clearance of polyubiquitinated proteins, p62 is activated when phosphorylated by TBK1. We found that the coexpression of TBK1, p62, and BinCARD1 led to a dramatic increase in p62 phosphorylation, LC3II induction and BinCARD1 degradation, thereby confirming that the degradation of BinCARD1 was mediated via the TBK1-p62 axis. These results highlight the complexity of the host cellular network in the regulation of IAV infection: the promoting role played by BinCARD1 in the nuclear import of the vRNP complex and replication of IAV was negatively regulated by selective autophagic degradation of BinCARD1 via the TBK1-p62 axis.

**Fig. 9:**
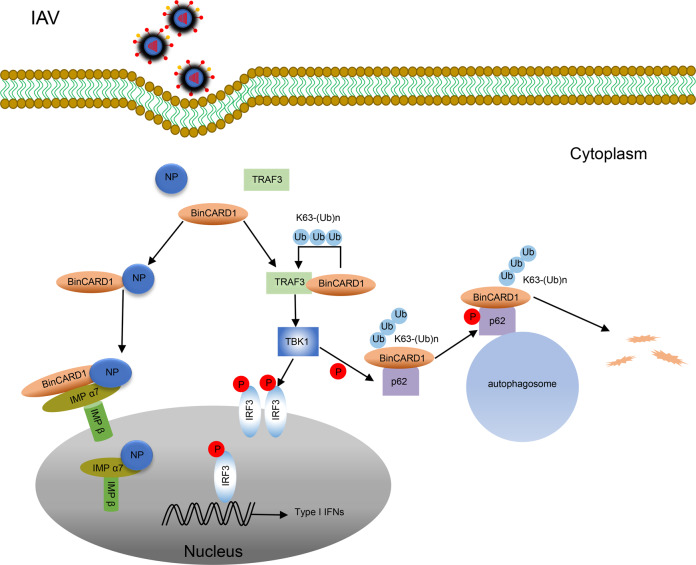
Schematic model showing the role of BinCARD1 in the replication cycle of IAV. BinCARD1 interacts with IAV NP and enhances the interaction between NP and IMP α7, thereby facilitating the nuclear import of the vRNP complex and newly synthesized NP. In contrast, the role played by BinCARD1 in promoting IAV replication is counteracted by the host defense system: BinCARD1-mediated Lys63-linked polyubiquitination of TRAF3 favors the activation of the RIG-I innate immune signaling pathway, and polyubiquitinated BinCARD1 is subjected to selective autophagic degradation via the TBK1-p62 axis

In summary, our study revealed that IAV engages BinCARD1, which is a positive regulatory host factor, to realize efficient viral replication. Mechanistically, BinCARD1 interacts with viral NP and enhances the interaction between NP and IMP α7, thereby facilitating the nuclear import of the vRNP complex and newly synthesized NP. However, the IAV leverage of BinCARD1 for its own replication induces an antagonistic response by the host: the interaction between BinCARD1 and TRAF3 favors the activation of the RIG-I innate immune signaling pathway, and polyubiquitinated BinCARD1 is then subjected to selective autophagic degradation via the TBK1-p62 axis (Fig. 9). Our data thus highlight the complexity of host cellular networks in the regulation of the IAV replication cycle.

## Supplementary information

Figure S1

Figure S2

Figure S3

Figure S4

Figure S5

Figure S6

Figure S7

Figure S8

Figure S9

Supplementary Materials

## Data Availability

All data needed to evaluate the conclusions in this study are presented in this manuscript or the Supplementary Information. The materials described in this study are either commercially available or available upon reasonable request from the corresponding authors.
