# Supplementary figures and images for: Influenza A virus use of BinCARD1 to facilitate the binding of viral NP to importin α7 is counteracted by TBK1-p62 axis-mediated autophagy
Source: Cell Mol Immunol. 2022 Sep 2;19(10):1168–84. doi: 10.1038/s41423-022-00906-w (PMC9508095; doi:10.1038/s41423-022-00906-w)

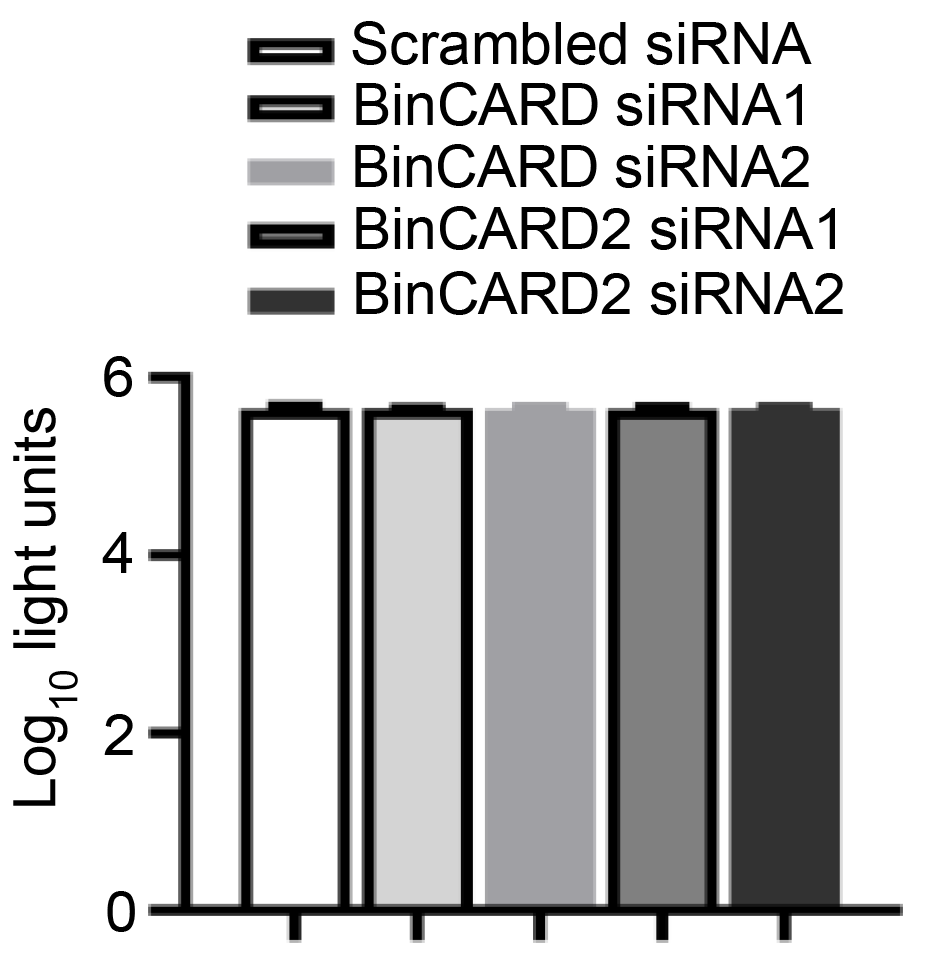

Supplement: Supplementary file 1 — Figure S1 [file 41423_2022_906_MOESM1_ESM.tif]

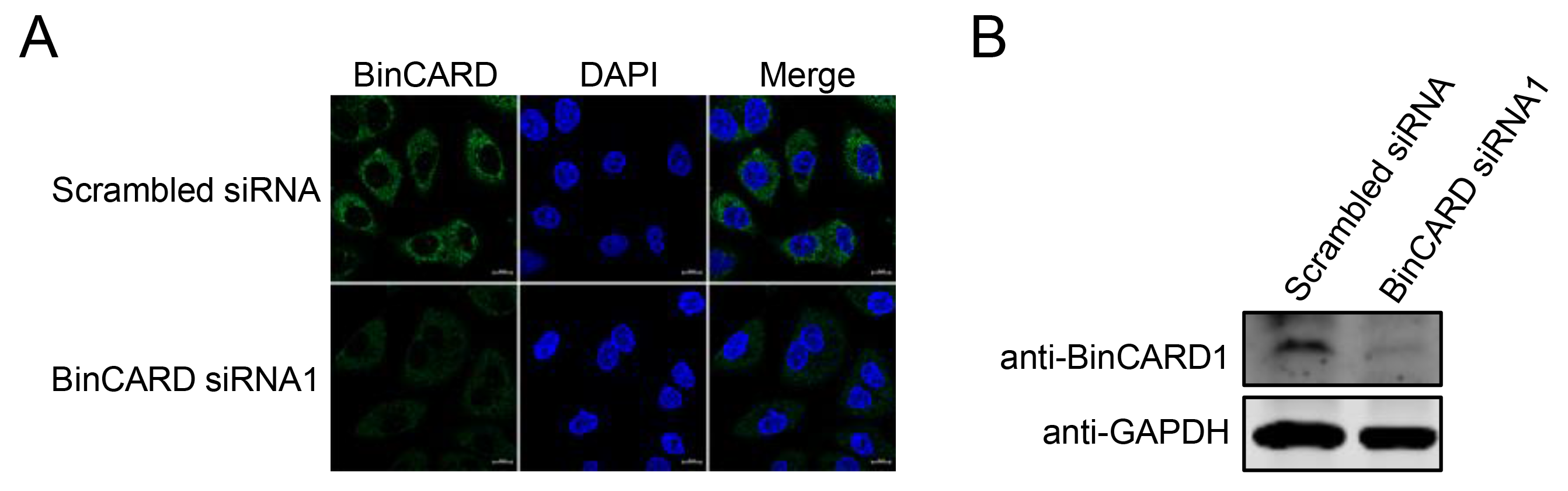

Supplement: Supplementary file 2 — Figure S2 [file 41423_2022_906_MOESM2_ESM.tif]

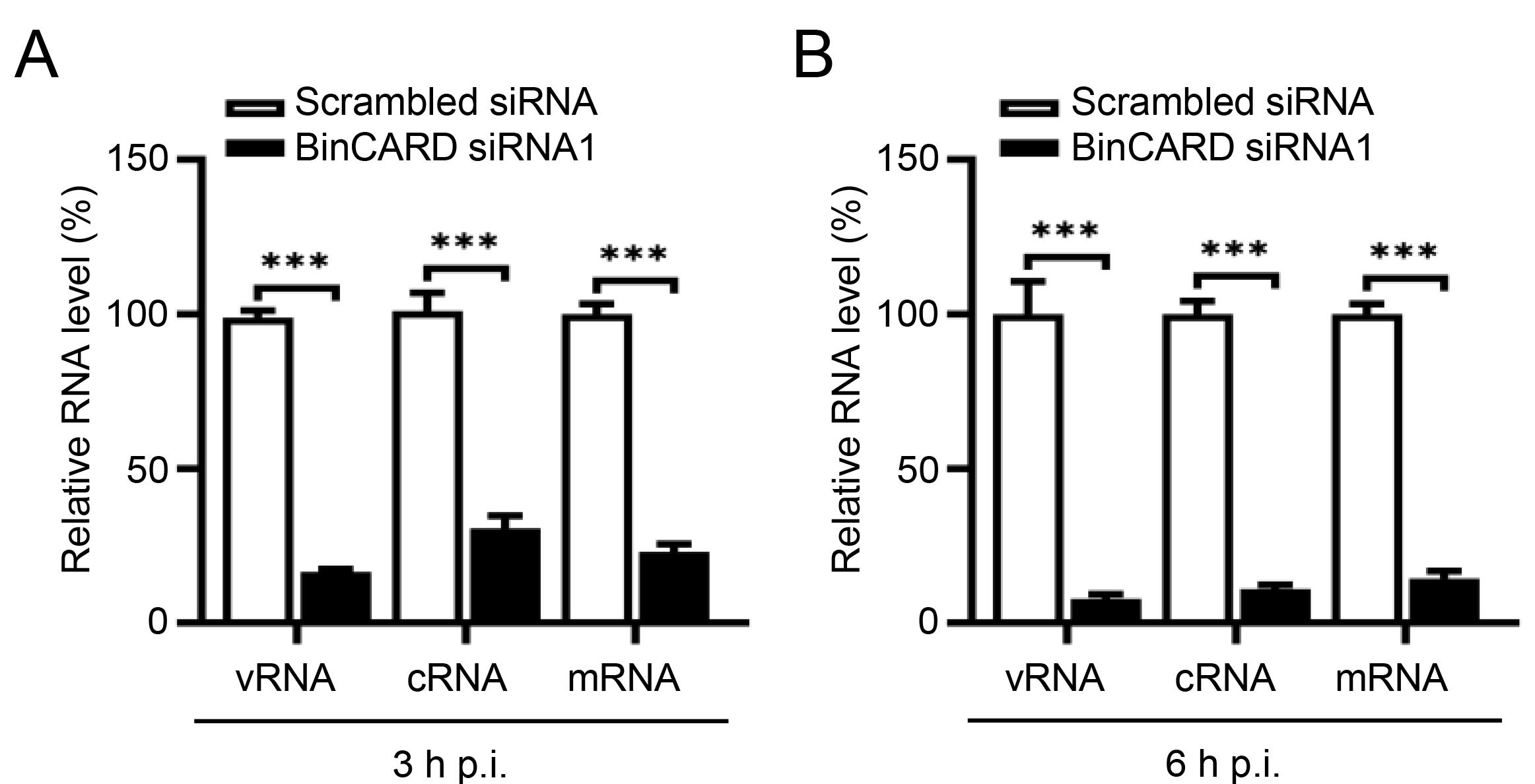

Supplement: Supplementary file 3 — Figure S3 [file 41423_2022_906_MOESM3_ESM.tif]

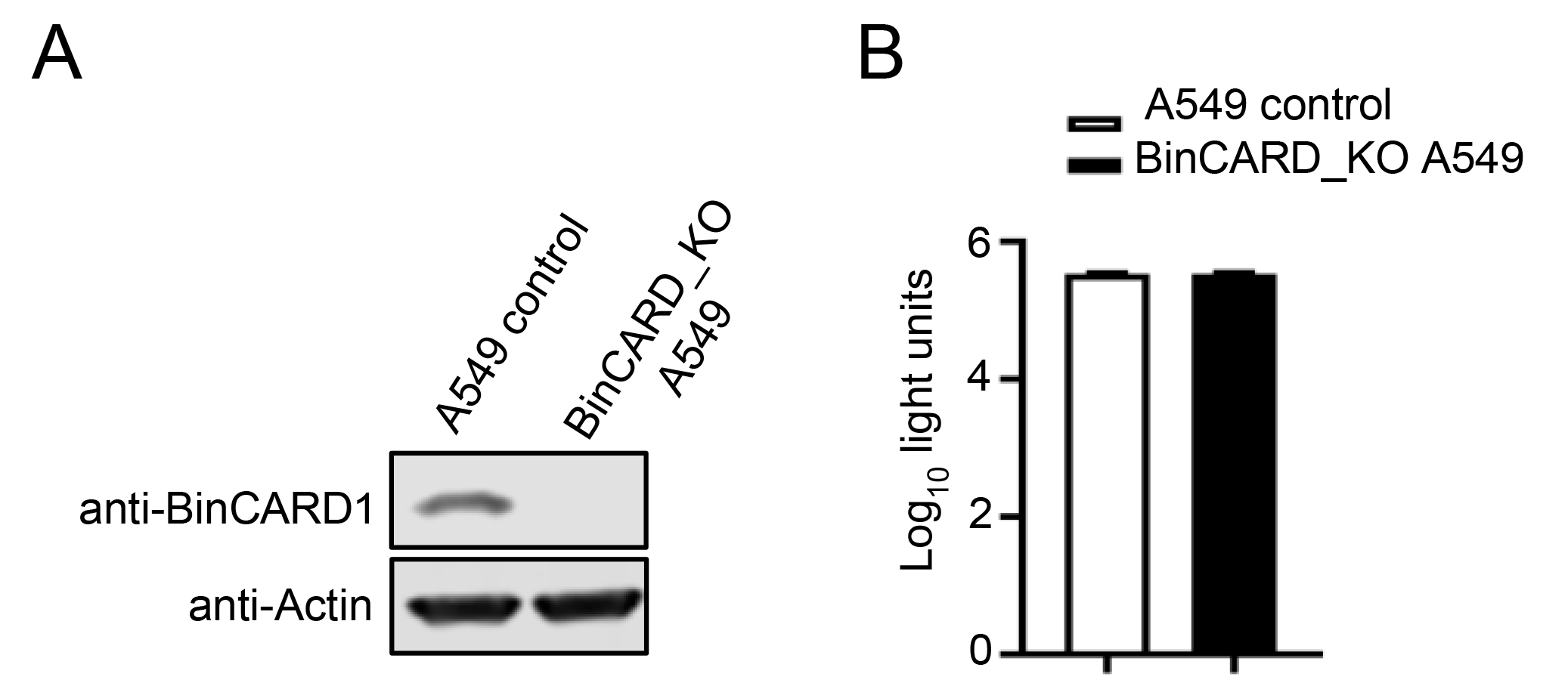

Supplement: Supplementary file 4 — Figure S4 [file 41423_2022_906_MOESM4_ESM.tif]

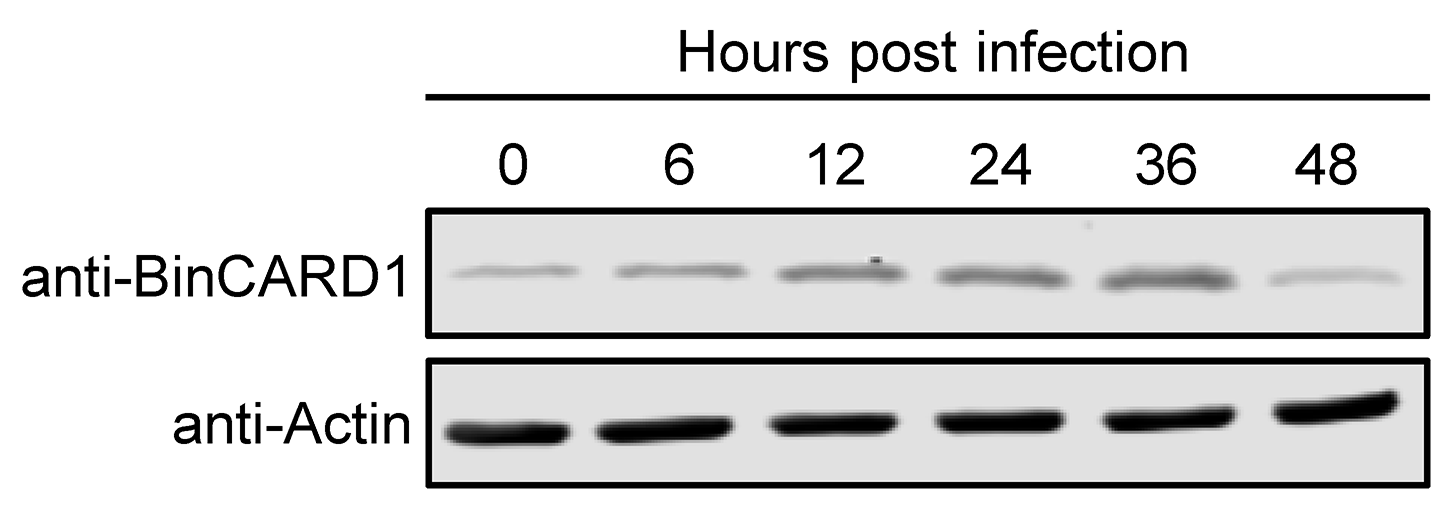

Supplement: Supplementary file 5 — Figure S5 [file 41423_2022_906_MOESM5_ESM.tif]

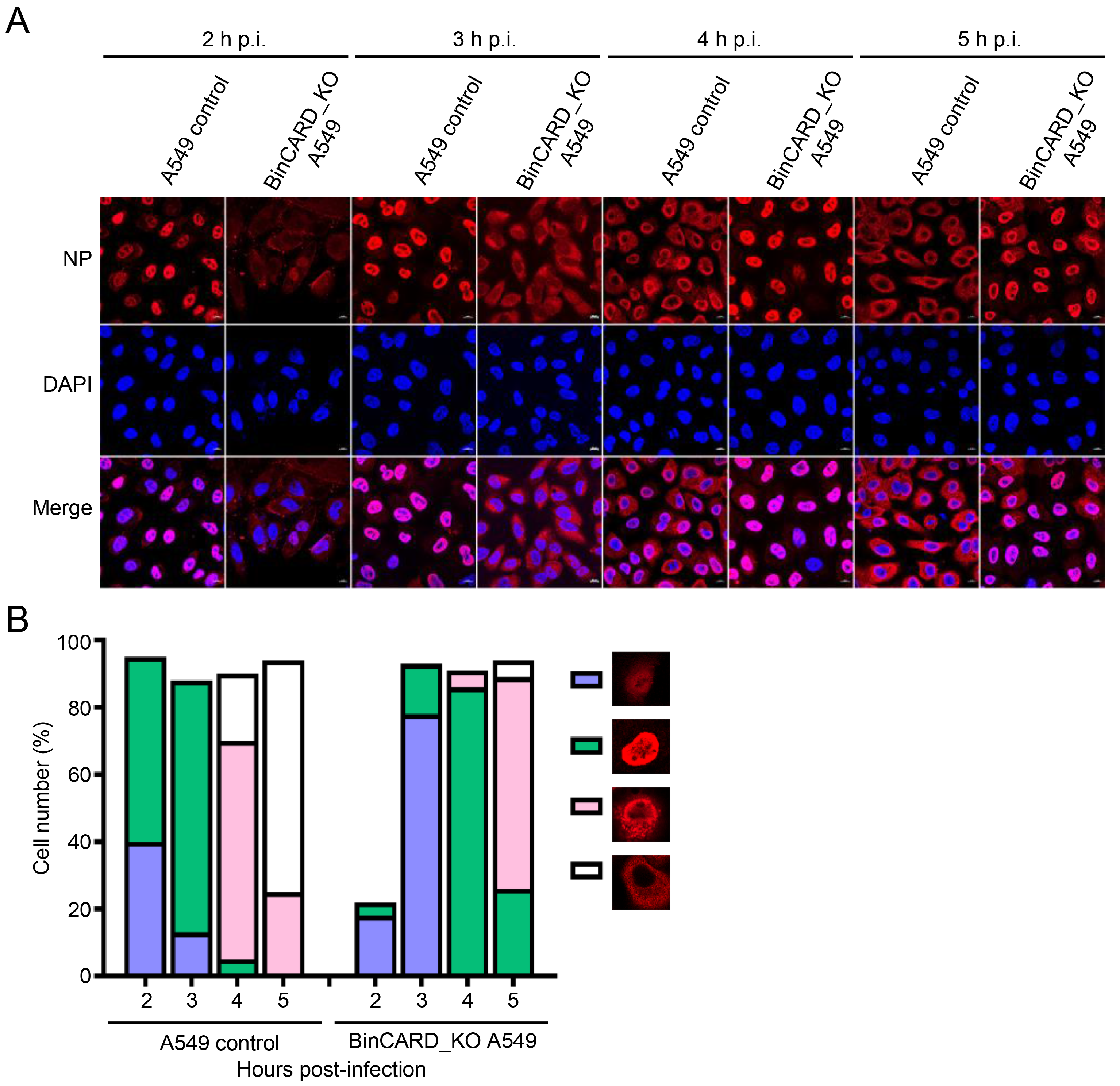

Supplement: Supplementary file 6 — Figure S6 [file 41423_2022_906_MOESM6_ESM.tif]

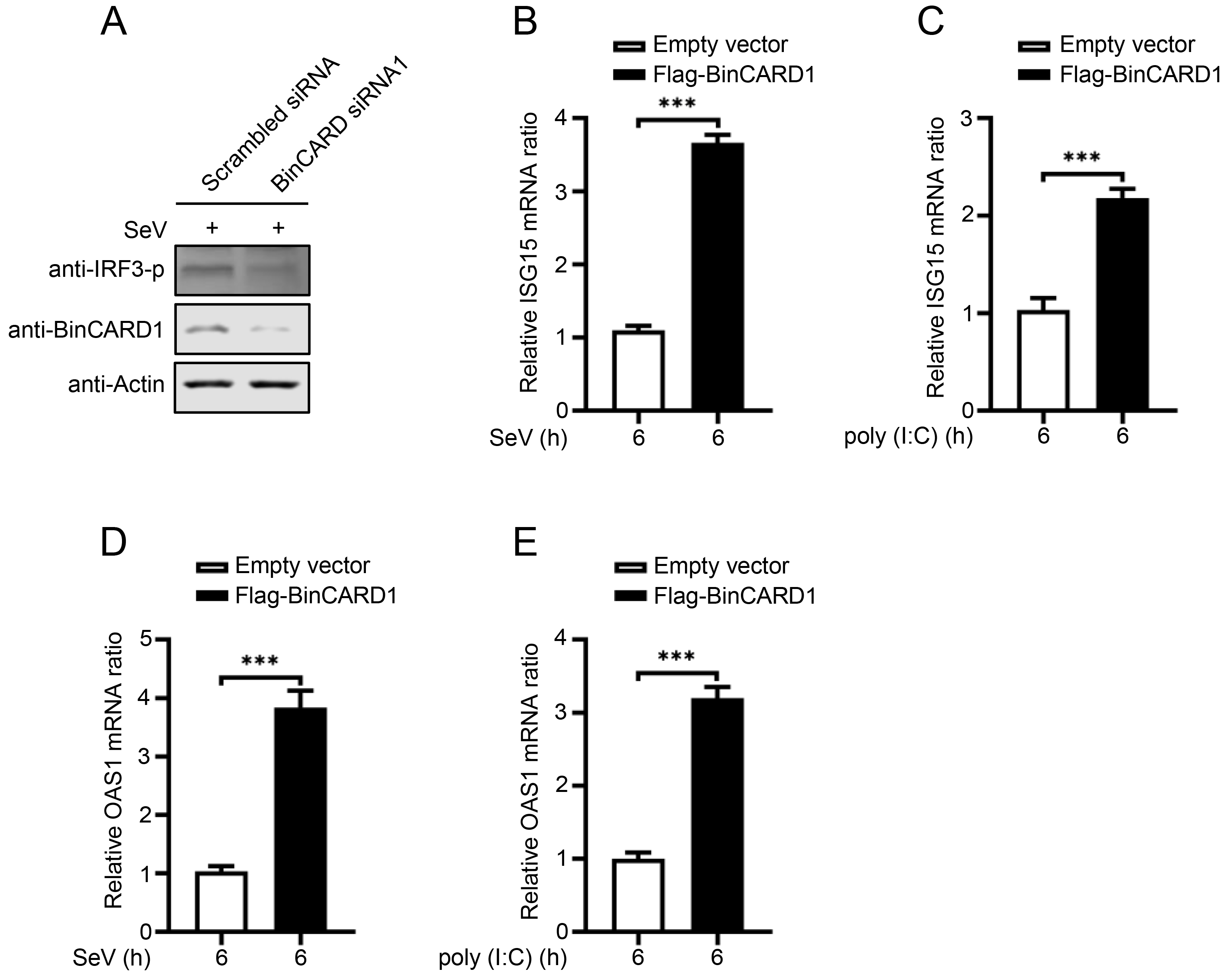

Supplement: Supplementary file 7 — Figure S7 [file 41423_2022_906_MOESM7_ESM.tif]

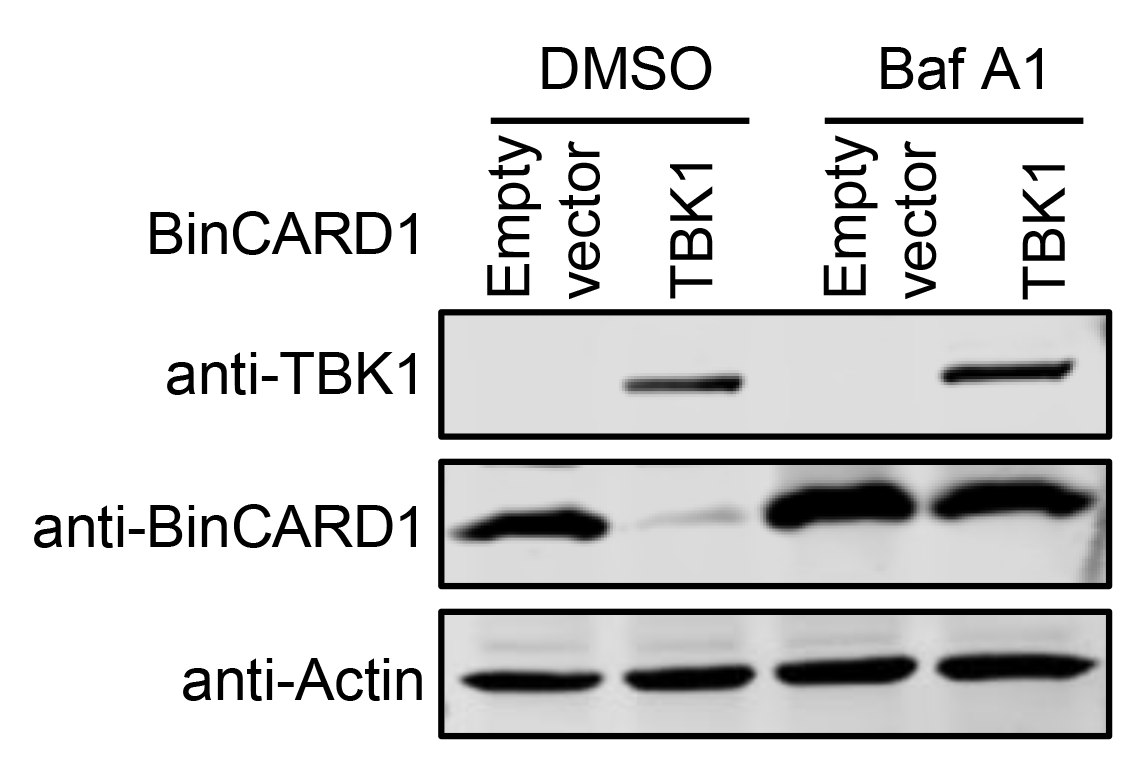

Supplement: Supplementary file 8 — Figure S8 [file 41423_2022_906_MOESM8_ESM.tif]

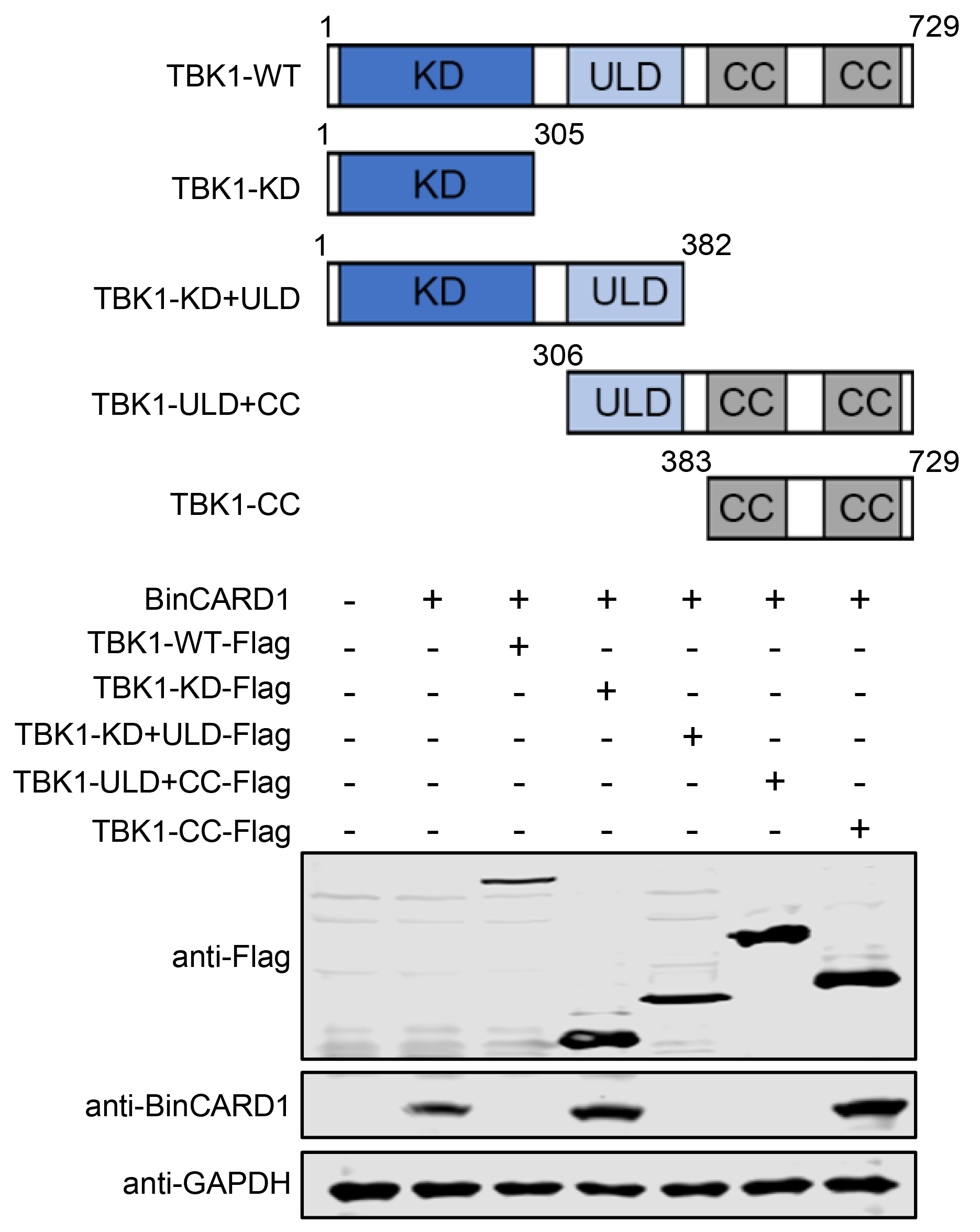

Supplement: Supplementary file 9 — Figure S9 [file 41423_2022_906_MOESM9_ESM.tif]
