# Supplementary material for: Influenza A virus use of BinCARD1 to facilitate the binding of viral NP to importin α7 is counteracted by TBK1-p62 axis-mediated autophagy
Source: Cell Mol Immunol. 2022 Sep 2;19(10):1168–84. doi: 10.1038/s41423-022-00906-w (PMC9508095; doi:10.1038/s41423-022-00906-w)
Supplement: Supplementary file 10 — Supplementary Materials [file 41423_2022_906_MOESM10_ESM.docx]

**Supporting Information**

**Fig. S1 Viability of BinCARD- or BinCARD2-siRNA-treated A549 cells.** A549 cells were transfected with siRNA targeting BinCARD or BinCARD2 or with scrambled siRNA at a concentration of 30 nM. At 36 h p.t., cell viability was determined by CellTiter-Glo assay.

**Fig. S2 Effect of BinCARD siRNA1 on BinCARD1 expression at the protein level.** (A, B) A549 cells were transfected with BinCARD siRNA1 or with scrambled siRNA. At 36 h p.t., the efficiency of knockdown of both BinCARD isoforms was determined by confocal microscopy with a rabbit anti-BinCARD pAb (A), and the efficiency of BinCARD1 knockdown was further assessed by western blotting with a mouse anti-BinCARD1 mAb (B).

**Fig. S3 Effect of BinCARD siRNA1 on the expression of the three viral RNA species of IAV.** (A, B) A549 cells were transfected with scrambled siRNA or BinCARD siRNA1, and at 36 h p.t., the cells were infected with WSN (H1N1) virus (MOI=5). At 3 (A) and 6 (B) h p.i., the levels of vRNA, mRNA, and cRNA derived from the NP gene were measured by RT‒qPCR. ***, *P* < 0.001.

**Fig. S4 Viability of BinCARD_KO A549 cells.** (A) Western blotting was performed with a mouse anti-BinCARD1 mAb to validate the knockout of BinCARD1 in BinCARD_KO A549 cells. (B) The viability of BinCARD_KO A549 cells was determined by CellTiter-Glo assay.

**Fig. S5 Expression profile of BinCARD1 in the course of IAV infection.** A549 cells were infected with WSN (H1N1) virus (MOI=0.01), and the expression of BinCARD1 was measured by western blotting with a mouse anti-BinCARD1 mAb at the indicated timepoints p.i.

**Fig. S6 Effect of BinCARD1 knockout on the cellular localization of NP during IAV infection as determined by confocal microscopy.** (A) BinCARD_KO A549 cells and A549 control cells were infected with WSN (H1N1) (MOI=5) virus. At 2, 3, 4, and 5 h p.i., the infected cells were fixed and stained with a mouse anti-NP mAb and were then incubated with Alexa Fluor 633 goat anti-mouse IgG (H+L) (red). The nuclei were stained with DAPI. (B) Quantitative analysis of NP localization in virus-infected BinCARD_KO A549 cells. On the basis of the confocal microscopy images in (A), the localization of NP (indicative of vRNP localization) upon the appearance of its nuclear localization was divided into the following categories: weak nuclear localization, strong nuclear localization, simultaneous localization at the boundary of the nucleus and the cytoplasm, or predominant cytoplasmic localization. The data shown were derived from 100 cells visualized by confocal microscopy with a 40X objective lens.

**Fig. S7 Effect of BinCARD1 on the activation of RIG-I signaling.** (A) Western blotting was performed to examine the effect of BinCARD1 on IRF3 phosphorylation. A549 cells were transfected with scrambled siRNA or BinCARD siRNA1, and at 36 h p.t., the cells were stimulated with SeV for 12 h. Cell lysate proteins were subjected to western blotting with a rabbit anti-IRF3-p mAb and a mouse anti-BinCARD1 mAb. (B-E) RT‒qPCR assays were performed to evaluate the effect of BinCARD1 on the levels of IFN-stimulated genes. HEK293T cells were transfected with a Flag-tagged BinCARD1-expressing plasmid or an empty vector, and at 36 h p.t., the cells were stimulated with SeV or poly (I:C) for 6 h. The mRNA levels of ISG15 (B, C) and OAS1 (D, E) in the cell lysates were measured by RT‒qPCR analysis. ***, *P* < 0.001.

**Fig. S8 Effect of bafilomycin A1 (Baf A1) treatment on TBK1-mediated degradation of BinCARD1.** HEK293T cells were transfected with plasmids expressing BinCARD1, and 12 h later, the cells were transfected with an empty vector or a plasmid expressing TBK1. Twenty-four hours later, the cells were treated for 8 h with DMSO or Baf A1. Cell lysate proteins were subjected to western blotting with a mouse anti-BinCARD1 mAb or a rabbit anti-TBK1 mAb.

**Fig. S9 The critical domain in TBK1 for mediating the degradation of BinCARD1 was determined by western blotting**. HEK293T cells were transfected with plasmids expressing BinCARD1 and Flag-tagged truncation mutants of TBK1. At 36 h p.t., cell lysate proteins were subjected to western blotting with a mouse anti-BinCARD1 mAb or a rabbit anti-Flag pAb.
